# RNA-binding motif protein 10 inactivates c-Myc by partnering with ribosomal proteins uL18 and uL5

**DOI:** 10.1073/pnas.2308292120

**Published:** 2023-11-30

**Authors:** Hyemin Lee, Ji Hoon Jung, Hyun Min Ko, Heewon Park, Allyson M. Segall, Roger L. Sheffmaker, Jieqiong Wang, Wesley D. Frey, Nathan Pham, Yongbo Wang, Yiwei Zhang, James G. Jackson, Shelya X. Zeng, Hua Lu

**Affiliations:** ^a^Department of Biochemistry and Molecular Biology, Tulane University School of Medicine, New Orleans, LA 70112; ^b^Tulane Cancer Center, Tulane University School of Medicine, New Orleans, LA 70112; ^c^Department of Neuroscience, Tulane University, New Orleans, LA 70118; ^d^Department of Cell and Molecular Biology, Tulane University, New Orleans, LA 70118; ^e^Department of Cellular and Genetic Medicine, School of Basic Medical Sciences, Fudan University, Shanghai 200032, China

**Keywords:** RBM10, Ribosomal protein L5/uL18, c-Myc, lung cancer, Ribosomal protein L11/uL5

## Abstract

RNA-binding motif protein 10 (RBM10) is a frequently mutated tumor suppressor in lung adenocarcinoma (LUAD). Yet, it remains unknown whether cancer-derived mutant RBM10 compromises its tumor suppression function and, if so, the molecular insight of the underlying mechanisms. Here, we show that wild-type RBM10 suppresses lung cancer cell growth and proliferation by inactivating c-Myc that is essential for cancer cell survival. RBM10 directly binds to c-Myc and promotes c-Myc’s ubiquitin-dependent degradation, while RBM10 knockdown leads to the induction of c-Myc level and activity. This negative action on c-Myc is further boosted by ribosomal proteins (RPs) uL18 (RPL5) and uL5 (RPL11) via their direct binding to RBM10. Cancer-derived mutant RBM10-I316F fails to bind to uL18 and uL5 and to inactivate c-Myc, thus incapable of suppressing tumorigenesis. Our findings uncover RBM10 as a pivotal c-Myc repressor by cooperating with uL18 and uL5 in lung cancer cells, as its failure to do so upon mutation favors tumorigenesis.

## Significance

Lung cancer is one of the most common cancers and the leading cause of cancer-related deaths in the world. Understanding the molecular mechanisms underlying lung cancer growth and progression is critical for developing early diagnosis and strategies for cancer prevention and therapy. This study reveals that the tumor suppressor RBM10 works with ribosomal proteins and acts to inhibit the oncogenic activity of c-Myc, whereas a lung cancer–derived RMB10 mutant fails to do so; instead, it promotes lung cancer growth. These findings not only unveil a mechanism underlying the action of this tumor suppressor in inhibiting lung cancer growth but also suggest a pathway for developing potential combinatorial therapies against malignant lung cancer.

c-Myc is essential for the growth and proliferation as well as drug resistance of cancer and cancer stem cells (1). These functions are primarily attributed to c-Myc’s transcriptional regulation of 10 to 15% of the genome, including proteins, both ribosomal and nonribosomal, and noncoding, regulatory RNAs. c-Myc is involved in cell division, death, survival, migration, metabolism, ribosome biogenesis, and immune response (2–10). Deregulation of c-Myc is highly associated with a wide range of cancers (5, 6). Amplification of the c-Myc-encoding gene is correlated with poor clinical outcome and tumor aggressiveness (11–14). Often, tumor cells that express high levels of c-Myc are no longer dependent on growth factor stimulation; this is different from normal cells, as growth stimulation is required for their c-Myc-dependent proliferation, metabolic pathways, and ribosome biogenesis (2, 15). Hence, controlling c-Myc expression and activity is critical for preventing cancer cell growth, proliferation, and drug resistance.

One of the cellular mechanisms underlying c-Myc regulation is negative feedback by ribosomal proteins L5 (RPL5/uL18) and L11 (RPL11/uL5) (7, 8, 10, 16, 17). Since the coordination of ribosomal biogenesis with protein translation is essential for healthy proliferation as well as for cancer growth and proliferation, this process is tightly regulated by a number of tumor suppressors and oncoproteins including c-Myc (16, 18). c-Myc enhances the transcription of many ribosomal biogenesis-related genes (17). As transcriptional targets of c-Myc, uL18, and uL5 can suppress c-Myc activity by enhancing c-Myc degradation in a negative feedback fashion (7, 16, 17). To understand how these RPs actions are regulated, we identified RNA-binding motif protein 10 (RBM10) as one of the uL18-binding proteins via coimmunoprecipitation coupled with mass spectrometric analysis (19). Our further investigation revealed that RBM10 partners with the RPs regulate c-Myc stability and activity as described below.

RBM10 is an RNA-binding protein frequently deleted or mutated in lung cancers as well as other types of cancers (20–22) and even homozygously deleted (Homdel) in some cancers (*SI Appendix*, Fig. S2). Recent reports showed that knockdown of RBM10 in human cancer cells enhances cell proliferation, suggesting that RBM10 acts as a tumor suppressor (23–25). RBM10 expression levels in LUAD patients as analyzed by RNA seq were lower than that in normal tissues (26) (*SI Appendix*, Fig. S1*A*). Also, the survival rate after initial diagnosis of metastatic non-small-cell lung cancer patients harboring RBM10 mutants was lower than that with wild-type RBM10 (27, 28) (*SI Appendix*, Fig. S1*B*), suggesting that RBM10 mutants deserve more attention. Most of the previous studies have focused on RBM10 as a splicing factor (24, 29–32). Our recent study showed that RBM10 can also suppress cancer cell growth and proliferation by activating p53 (20, 33). Hence, identification of RBM10 in our present study as another regulator of c-Myc by partnering with uL18 and uL5 would unveil a function of this tumor suppressor.

Here, we report that RBM10 can inhibit c-Myc activity without affecting its pre-mRNA splicing. We found that overexpression of RBM10 markedly inhibits c-Myc expression in cancer cells by destabilizing c-Myc protein via ubiquitin-mediated proteolysis. Interestingly, this inhibition is dependent upon uL18 and uL5. Strongly supporting this, the lung cancer–derived RBM10 mutant, RBM10-I316F, fails to bind to uL18 or uL5, to suppress c-Myc activity, and thus to inhibit lung cancer growth. Instead, this mutant appears to further augment lung cancer growth. Taken together, our results demonstrate that RBM10 inhibits c-Myc expression and activity by interacting with uL18 or uL5.

## Results

### RBM10 Suppresses Serum-Responsive c-Myc Expression, Independent of Splicing Regulation.

**Fig. 1.**
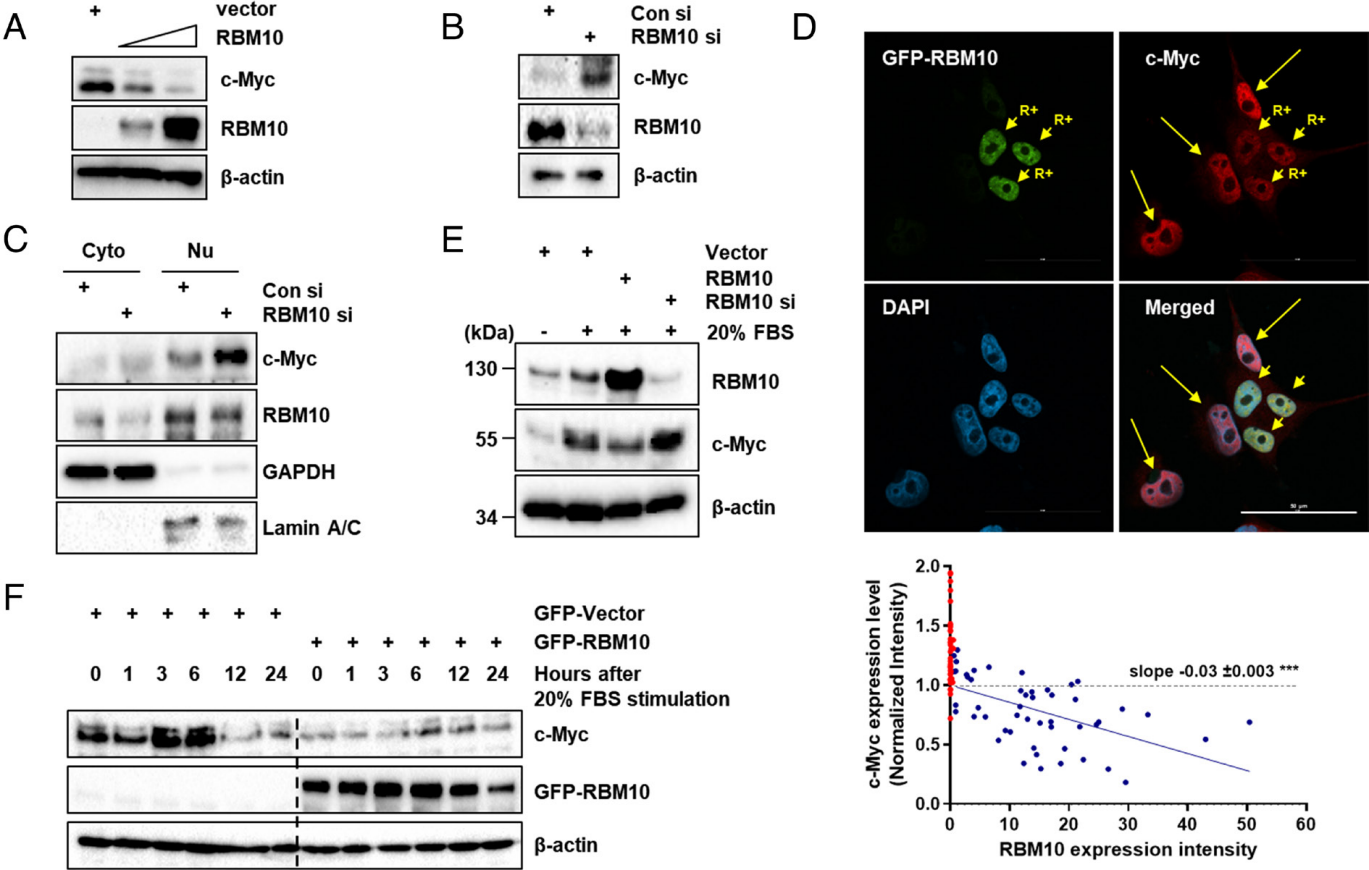
RBM10 inhibits c-Myc expression. (*A*) HCT116^p53−/−^ cells were transfected with control vector or RBM10 plasmids in different amounts as indicated and harvested 48 h post transfection. Proteins were analyzed by IB. (*B*) HCT116^p53−/−^ cells were transfected with control or RBM10 siRNA and harvested 72 h post transfection for IB analysis with indicated antibodies. (*C*) HCT116^p53−/−^ cells were transfected with control or RBM10 siRNA and harvested 72 h post transfection for cytoplasmic and nuclear extraction and IB analysis. GAPDH was used for cytoplasm loading control. Lamin A/C was used for nuclear loading control. (*D*) GFP-RBM10 was overexpressed in H1299 cells for 48 h, fixed, and permeabilized. The samples were stained with α-c-Myc following with Alexa-594 secondary antibody and detected using confocal microscopy (*Top*). Short arrows with “R+” indicate GFP-RBM10 overexpressing cells. Long arrows indicate cells without overexpression of GFP-RBM10. DAPI was used to confirm nucleus localization. (Scale bar, 50 μm.) The c-Myc expression level was normalized (*Bottom*). Each cell was described as a dot (red: no RBM10 overexpression; blue: GFP-RBM10 overexpressed). (*E*) HCT116^p53−/−^ cells were transfected with control vector, RBM10 siRNA, or RBM10 overexpressing plasmid for 48 h, and the cells were starved in 0.2% serum for 30 h and stimulated with 20% serum for 6 h. The protein levels were measured by IB. (*F*) HCT116^p53−/−^ cells were transfected with GFP-tagged control vector or RBM10. The cells were treated with serum starvation and stimulation with 20% FBS treatment and harvested at indicated time points. Proteins were analyzed by IB with indicated antibodies. GFP-RBM10 was detected using the α-GFP antibody.

Our recent study showed that RBM10 can suppress cell proliferation and survival in p53-null colorectal cancer, even though it also does so by activating p53 (20, 33). In our attempt to address the p53-independent tumor suppression function of RBM10, we found that RBM10 can decrease the protein level of c-Myc, as overexpression of RBM10 led to the drastic decrease of c-Myc protein levels in p53-deficient colorectal cancer HCT116 (Fig. 1*A*) and lung cancer H1299 cells (*SI Appendix*, Fig. S3*A*). Consistently, knockdown of RBM10 led to the increase of c-Myc protein levels in HCT116^p53−/−^ cells (Fig. 1*B*). However, overexpression of c-Myc did not markedly affect the RBM10 expression level (*SI Appendix*, Fig. S3 *B* and *C*). Most of RBM10 and c-Myc molecules localized to the nucleus as detected by immunofluorescence (IF) staining (*SI Appendix*, Fig. S4 *A*–*C*). To test where RBM10 affects c-Myc protein level, we performed biochemical fractionation (Fig. 1*C*) and IF assays (Fig. 1*D*). We found that knockdown of RBM10 increases nuclear c-Myc level (Fig. 1*C*), and overexpression of RBM10 reduces c-Myc level in the nucleus (Fig. 1*D*). Because c-Myc is rapidly induced in response to serum stimulation (34), we next determined whether this serum-responsive induction could be affected by RBM10 by introducing either ectopic RBM10 or RBM10 siRNA into HCT116^p53−/−^ cells. HCT116^p53−/−^ cells were serum-starved for 30 h and then cultured in media containing 20% fetal bovine serum (FBS). Cells were harvested at 6 h after serum stimulation for immunoblotting (IB) analysis. As expected, the induction of c-Myc expression was dramatically reduced in cells with RBM10 overexpression (Fig. 1*E*, lane 3 vs. lane 2). In contrast, c-Myc was induced in cells, in which RBM10 was knocked down, compared with serum-stimulated control (lane 4 vs. lane 2) (Fig. 1*E*). Consistently, ectopic RBM10 markedly reduced serum-responsive c-Myc expression even at the 6-h peak after serum treatment (Fig. 1*F*).

Since RBM10 was previously shown to inhibit cancer cell proliferation via its effect on alternative splicing (24), we tested whether RBM10 might reduce c-Myc level by regulating its RNA splicing. First, we checked the mRNA level of c-Myc after RBM10 was altered. As shown in *SI Appendix*, Fig. S5 *A* and *B*, neither overexpression nor knockdown of RBM10 altered c-Myc mRNA level or c-Myc RNA splicing variants. As expected (35), the splicing of Fas pre-mRNAs was affected by RBM10 under the same conditions (left columns of *SI Appendix*, Fig. S5 *A* and *B*). Interestingly, RBM10 still reduced c-Myc protein levels (*SI Appendix*, Fig. S5*C*) even when lung cancer cells were treated with Isoginkgetin, a small molecule inhibitor of pre-mRNA splicing (36). Altogether, these results demonstrate that RBM10 inhibits serum-responsive c-Myc expression at its protein level in the nucleus without affecting c-Myc pre-mRNA alternative splicing.

### RBM10 Interacts with c-Myc.

**Fig. 2.**
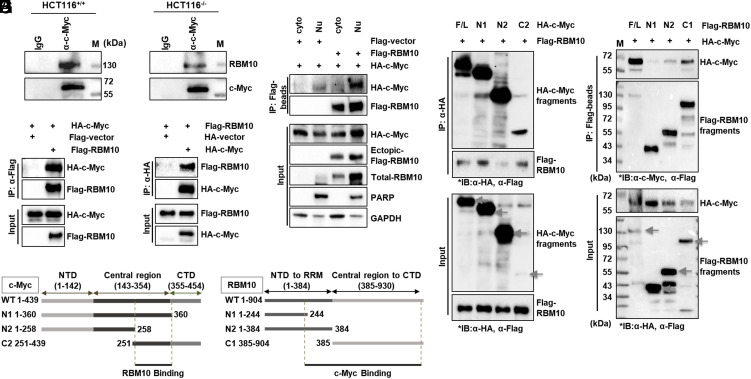
RBM10 interacts with c-Myc. (*A* and *B*) The association between endogenous RBM10 and c-Myc is detected in HCT116^p53+/+^ and HCT116^p53−/−^ cells by co-IP-IB assays using antibodies as indicated. IgG was used as a control. α-c-Myc and α-RBM10 were used for IB detection. (*C* and *D*) Exogenous RBM10 interacts with exogenous c-Myc. HCT116^p53−/−^ cells were transfected with plasmids encoding HA-c-Myc and Flag-vector of Flag-RBM10 followed by co-IP-IB assays using antibodies. α-HA and α-Flag were used for IB detection. (*E*) After HCT116^p53−/−^ cells were transfected with HA-c-Myc plus Flag-control vector or Flag-RBM10 plasmids, cell fractionation assay was performed. The samples were incubated with Flag-Beads for immunoprecipitation. Input and the bound proteins were detected by IB. α-HA, α-Flag, α-RBM10, α-PARP, and α-GAPDH were used for IB detection. PARP and GAPDH were used for nuclear and cytosol loading controls. (*F* and *G*) Mapping the RBM10 binding domain of c-Myc by co-IP-IB analysis. HCT116^p53−/−^ cells were transfected with an RBM10-encoded plasmid along with the plasmid encoding each individual HA-c-Myc fragment as shown Fig. 2*F*. Co-IP assays were performed using the anti-HA antibody followed by IB with indicated antibodies. (*H* and *I*) Mapping the c-Myc binding domain of RBM10 by co-IP-IB analysis. H1299 cells were cotransfected with Flag-RBM10 fragments plasmids and HA-c-Myc plasmids. IB was followed with indicated antibodies after co-IP assay using Flag-beads was performed.

In order to elucidate the mechanisms underlying the RBM10 suppression of c-Myc protein expression, we first checked whether RBM10 might bind to c-Myc in the nucleus by performing coimmunoprecipitation (co-IP)-IB assays. Indeed, endogenous RBM10 was pulled-down with endogenous c-Myc by the c-Myc antibody in HCT116^p53+/+^ and HCT116^p53−/−^ cells (Fig. 2 *A* and *B*). This association was further verified by overexpressing both HA-c-Myc and Flag-RBM10 in HCT116^p53−/−^ cells followed by inverse co-IP assays with either anti-Flag or anti-HA antibodies (Fig. 2 *C* and *D*). To validate whether their association is a direct interaction, we used His-tagged RBM10 proteins purified from *E*scherichia* coli* as bait for nickel-bead pull-down assays. Both endogenous c-Myc in HCT116^p53+/+^ cells and ectopic c-Myc in HCT116^p53−/−^ cells bound to His-tagged RBM10 proteins (*SI Appendix*, Fig. S6 *A* and *B*). To determine where RBM10 and c-Myc interactions occur in the cells, we conducted cell fractionation and co-IP-IB assays and found that Flag-RBM10 and HA-c-Myc form complexes in the nucleus (Fig. 2*E*). To map their binding domains, we also performed co-IP-IB assays after cotransfecting HCT116^p53−/−^ cells with Flag-RBM10 and HA-c-Myc fragments (Fig. 2 *F* and *G*) or with HA-c-Myc and Flag-RBM10 fragments (Fig. 2 *H* and *I*). As shown in Fig. 2 *F* and *G*, RBM10 bound to the central domain of c-Myc. Because c-Myc dimerizes with Max to bind to DNA as a transcription factor (37), we tested whether RBM10 might influence the c-Myc-Max interaction by binding to this domain. We cotransfected c-Myc, Max and RBM10 in HCT116^p53−/−^ cells followed by a co-IP-IB assays. As a result, RBM10 did not affect formation of the c-Myc-Max complex (*SI Appendix*, Fig. S7). As shown in Fig. 2 *H* and *I*, c-Myc primarily bound to the C terminus of RBM10. Taken together, these results indicate that the C terminus of RBM10 interacts with the central domain of c-Myc in the nucleus.

### RBM10 Enhances c-Myc Ubiquitination and Proteolytic Degradation.

**Fig. 3.**
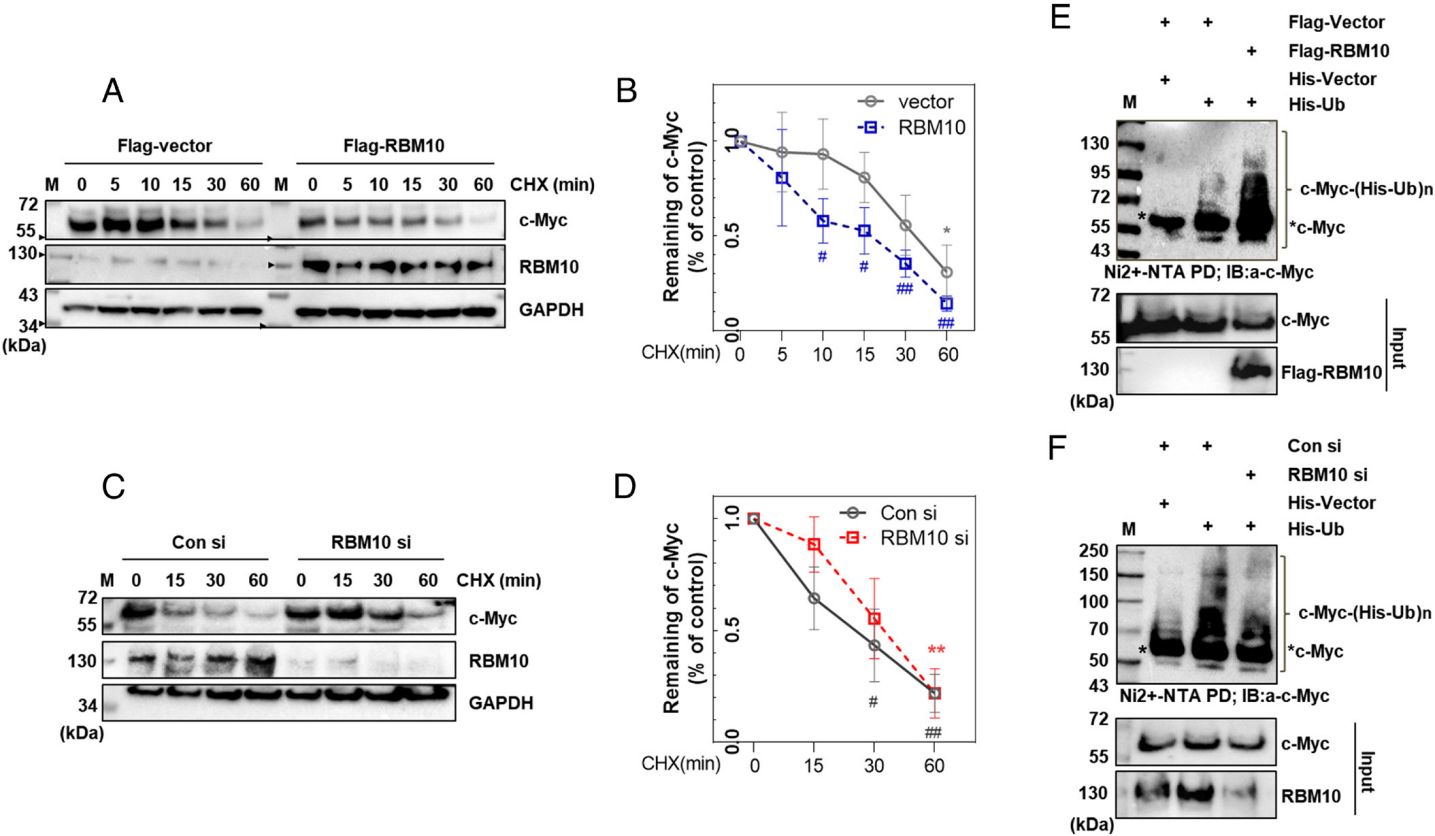
RBM10 regulates c-Myc protein stability. (*A*) c-Myc’s half-life is decreased upon RBM10 overexpression. HCT116^p53−/−^ cells were treated with Flag-control vector or Flag-RBM10 overexpressed plasmid for 48 h, then treated with 100 µg/mL of CHX (cycloheximide), and harvested at different time points as indicated for IB analysis (of note, shorter exposure for the RBM10 blot). (*B*) c-Myc protein expression level for Fig. 3*A* was measured using Image J after three independent experiments were performed. (*C*) c-Myc’s half-life is increased upon RBM10 knockdown. HCT116^p53−/−^ cells were treated with scramble or RBM10 siRNA for 48 h, treated with 100 µg/mL of CHX, and harvested at different time points as indicated for IB analysis. (*D*) c-Myc protein expression level for Fig. 3*C* was measured using Image J after three individual experiments were performed. (*E*) HCT116^p53−/−^ cells were transfected with combinations of plasmids as indicated for overexpression, respectively. The cells were treated with MG132 (40 µM) for 6 h and harvested for a ubiquitination assay. (*F*) HCT116^p53−/−^ cells were transfected with scrambled or RBM10 siRNA together with or without His-Ub and treated with MG132 (40 µM) for 6 h. For both panels *E* and *F*, IB analysis using antibodies, α-c-Myc, α-RBM10, and α-Flag, was performed to detect bound and input proteins.

Next, we determined whether RBM10 might affect c-Myc protein stability. First, we tested the half-life of c-Myc in the presence of ectopic RBM10. As shown in Fig. 3 *A* and *B*, overexpression of RBM10 led to a drastic reduction of c-Myc’s half-life in HCT116^p53−/−^ cells, while knockdown of RBM10 extended the half-life of c-Myc significantly in HCT116^p53−/−^ cells (Fig. 3 *C* and *D*). Then, we determined whether RBM10 might affect c-Myc ubiquitylation by conducting ubiquitination assays in cells. Overexpression of RBM10 enhanced ubiquitination of both endogenous (Fig. 3*E*) and exogenous c-Myc (*SI Appendix*, Fig. S8*A*), while knockdown of RBM10 reduced ubiquitination of endogenous (Fig. 3*F*) and exogenous c-Myc (*SI Appendix*, Fig. S8*B*). We also detected that RBM10 bound to Fbw7α (F-box and WD repeat domain-containing 7), a subunit of E3 ubiquitin ligase responsible for degradation of c-MYC (38) (*SI Appendix*, Fig. S8*C*) and enhanced degradation of c-Myc by Fbw7α (*SI Appendix*, Fig. S8*D*). Consistently, RBM10 knockdown alleviated Fbw7α’s ability to degrade c-Myc (*SI Appendix*, Fig. S8*E*). These results demonstrate that RBM10 can destabilize c-Myc by enhancing its ubiquitination and degradation likely mediated by Fbw7α.

### uL18 and uL5 Are Required for RBM10 Suppression of c-Myc.

**Fig. 4.**
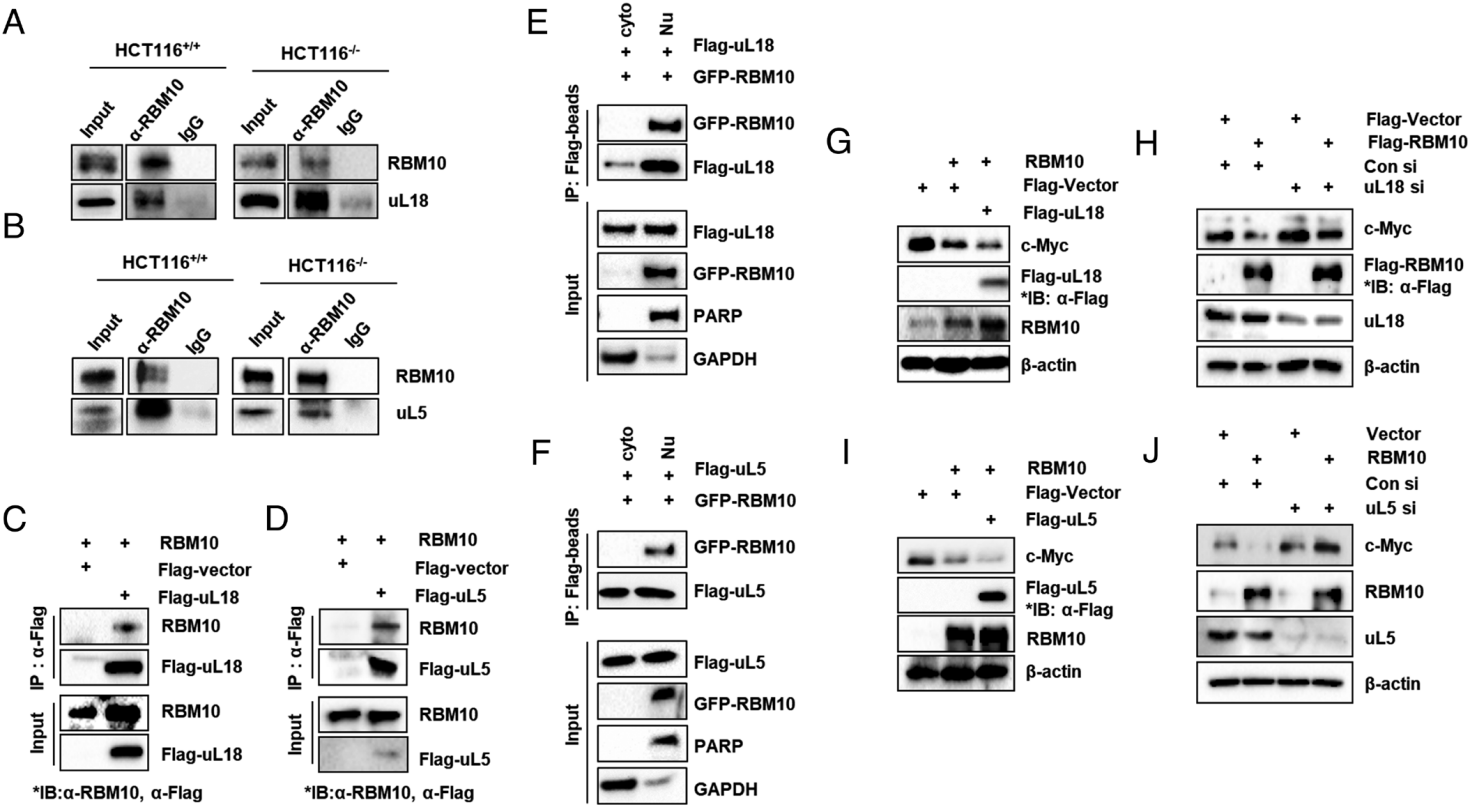
RBM10 interacts with uL18 or uL5 and regulates c-Myc depending on the RPs. (*A* and *B*) The association between endogenous RBM10 and uL18 (*A*) or uL5 (*B*) is detected in HCT116^p53+/+^ and HCT116^p53−/−^ cells by co-IP-IB assays using antibodies as indicated. α-IgG was used as a control. (*C*) Exogenous RBM10 interacts with uL18. HCT116^p53−/−^ cells were transfected with plasmids encoding RBM10 and Flag-uL18 (or Flag-vector for control) followed by co-IP-IB assays using antibodies as indicated. (*D*) Exogenous RBM10 interacts with uL5. HCT116^p53−/−^ cells were transfected with plasmids encoding RBM10 and Flag-uL5 (or Flag-vector for control) followed by co-IP-IB assays using antibodies as indicated. (*E* and *F*) After H1299 cells were overexpressed using Flag-uL18 (*E*) or Flag-uL5 (*F*) with GFP-RBM10 plasmids, cell fractionation assay was performed. co-IP assay using Flag-beads followed. The protein expression levels were confirmed by IB with α-GFP, α-Flag, α-PARP, and α-GAPDH. (*G* and *I*) HCT116^p53−/−^ cells were transfected with indicated plasmids and harvested 48 h post transfection. Proteins were analyzed by IB with indicated antibodies. (*H* and *J*) HCT116^p53−/−^ cells were transfected with uL18 (*H*), uL5 (*J*), or control siRNA for 24 h and then transfected with either Flag-control vector or Flag-RBM10 for 48 h. Proteins were analyzed by IB with indicated antibodies.

Previously, we reported that uL18/RPL5 and uL5/RPL11 can suppress c-Myc expression by directly binding to and destabilizing its protein (16, 34, 39). Thus, we tested whether RBM10 might regulate c-Myc expression via uL18 and/or uL5. First, we performed a set of co-IP-IB assays. Endogenous uL18 and uL5 were pulled down with endogenous RBM10 by RBM10 antibodies in HCT116^p53+/+^ and HCT116^p53−/−^ cells (Fig. 4 *A* and *B*). This interaction was further verified by overexpressing either RBM10 with Flag-uL18 or RBM10 with Flag-uL5 in HCT116^p53−/−^ cells followed by co-IP-IB assays (Fig. 4 *C* and *D*). Interestingly, the two protein complexes were detected in the nucleus by performing cell fractionation followed by co-IP-IB assays (Fig. 4 *E* and *F*). Endogenous 5s rRNA was also pulled down along with endogenous uL18 and uL5 as well as Flag-RBM10 by the anti-Flag antibody (*SI Appendix*, Fig. S9*A*). Furthermore, depletion of 5s rRNA drastically reduced the formation of the RBM10-uL18-uL5 complex (*SI Appendix*, Fig. S9*B*). These results demonstrate that 5s rRNA is essential for the formation of this RBM10-ribosomal protein complex.

Next, we tested whether uL18 or uL5 could synergize the suppressive effect of RBM10 on c-Myc. Interestingly, coexpression of RBM10 with either uL18 or uL5 in HCT116^p53−/−^ cells led to the further reduction of c-Myc protein levels (Fig. 4 *G* and *I*), while knockdown of either uL18 or uL5 impaired the RBM10 reduction of c-Myc protein levels in the cells (Fig. 4 *H* and *J*). Together, our results demonstrate that RBM10 suppression of c-Myc protein expression is dependent on uL18 and uL5, suggesting that RBM10 might execute this suppressive activity on c-Myc by forming a complex with 5s rRNA, uL18, and uL5.

### Cancer-Derived Mutant RBM10-I316F Fails to Suppress c-Myc Expression.

**Fig. 5.**
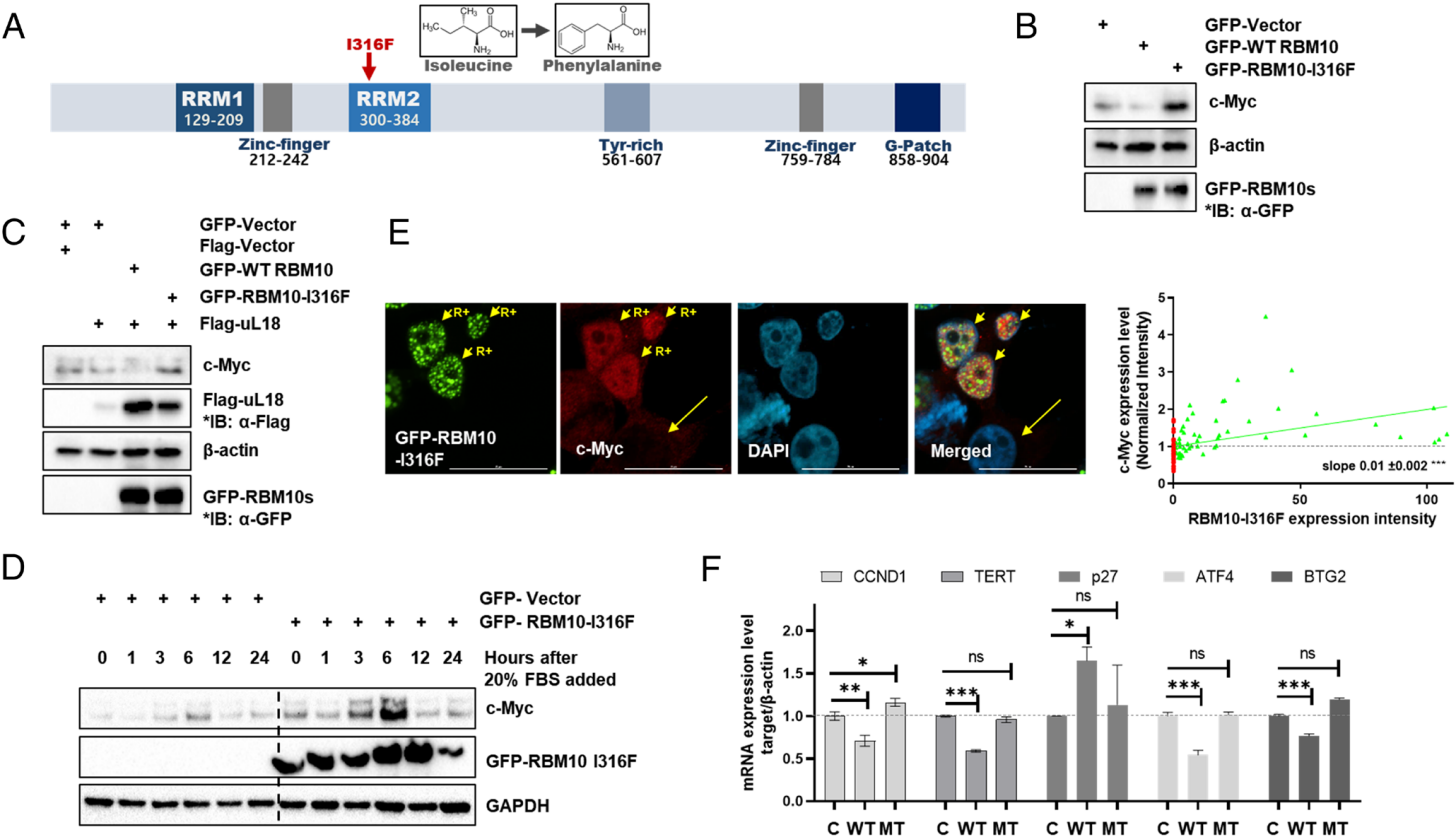
RBM10-I316F induces c-Myc expression. (*A*) Mapping the RBM10 domain (red arrow points I316F mutation). (*B*) HCT116^p53−/−^ cells were transfected with GFP-tagged control vector, RBM10, or RBM10-I316F encoded plasmid as indicated. Proteins were analyzed by IB with indicated antibodies. (*C*) HCT116^p53−/−^ cells were transfected with GFP-tagged RBM10 or RBM10-I316F encoded plasmid with Flag-uL18 or control vector as indicated. Proteins were analyzed by IB with indicated antibodies. (*D*) After HCT116^p53−/−^ cells were transfected with GFP-RBM10-I316F for 48 h, serum starvation was conducted for 36 h. The medium was changed to 20% FBS to stimulate c-Myc expression level. The protein levels were confirmed by IB after being harvested at time points indicated. (*E*) After H1299 cells were overexpressed with GFP-RBM10-I316F for 48 h, the cells were fixed, permeabilized, and stained with α-c-Myc followed by Alexa-594 secondary antibody staining. DAPI was used as an indicator of nuclei. Short arrows with “R+” indicate GFP-RBM10-I316F overexpressing cells. Long arrows indicate cells without overexpression of GFP-RBM10-I316F. (Scale bar, 25 μm.) The c-Myc expression level was normalized (*Right* panel). Each cell was described as a dot (red: no RBM10 overexpression; green: GFP-RBM10-I316F overexpressed). (*F*) After HCT116^p53−/−^ cells were transfected with GFP-tagged control, RBM10, or RBM10-I316F, RNAs were isolated and used for qRT-PCR for quantification of c-Myc’s downstream target genes as indicated. β-actin was used as loading control. C: GFP-control, WT: GFP-RBM10, MT: GFP-RBM10-I316F. The Student’s two-tailed *t* test was used to determine the mean difference among groups. Data are mean ± SD. *P < 0.05; **P < 0.01; and ***P < 0.001.

To illustrate which domain(s) or amino acid(s) of RBM10 would be a key for its suppression of c-Myc activation through uL18 and uL5, we employed the lung cancer–derived mutant RBM10-I316F (40) (Fig. 5*A*), which markedly increased lung adenocarcinoma (LUAD) A549 cell proliferation in cell culture (40). Interestingly, this mutant RBM10 also failed to decrease the protein level of endogenous c-Myc compared to wild-type RBM10 in HCT116^p53−/−^ cells, instead increasing the c-Myc level (Fig. 5*B*). While uL18 synergistically suppressed the c-Myc level when coexpressed with wild-type RBM10, coexpression of uL18 with RBM10-I316F did not suppress c-Myc in HCT116^p53−/−^ cells (Fig. 5*C*). Consistent with these results, this mutant RBM10 appeared to enhance serum-responsive expression of c-Myc at the protein level as detected by IB assays (Fig. 5*D*) and increased c-Myc level in the nucleus of HCT116^p53−/−^ cells as detected by IF analysis (Fig. 5*E*). Of note, the expression level of c-Myc protein was proportional to that of the RBM10-I316F in the cells (*Right* panel graph of Fig. 5*E*). Accordingly, the expression of c-Myc’s downstream target genes, such as CCND1, TERT, ATF4, and BTG2, was suppressed by wild-type RBM10, but not RBM10-I316F, whereas p27 mRNA level was up-regulated by wild-type RBM10, but not RBM10-I316F (Fig. 5*F*), indicating that the transcriptional activity of c-Myc is inhibited by wild-type RBM10, but not the mutant RBM10. Taken together, these results demonstrate that the cancer-derived mutant RBM10-I316F loses its ability to suppress c-Myc expression and activity. The results also suggest that this mutant RBM10 might gain an ability to increase c-Myc expression, implying a potentially dominant negative effect on its wild-type counterpart, as the mutant and wild-type RBM10s can bind to each other as confirmed by co-IP-WB analysis (*SI Appendix*, Fig. S10).

### RBM10-I316F Fails to Bind to uL18 and uL5.

**Fig. 6.**
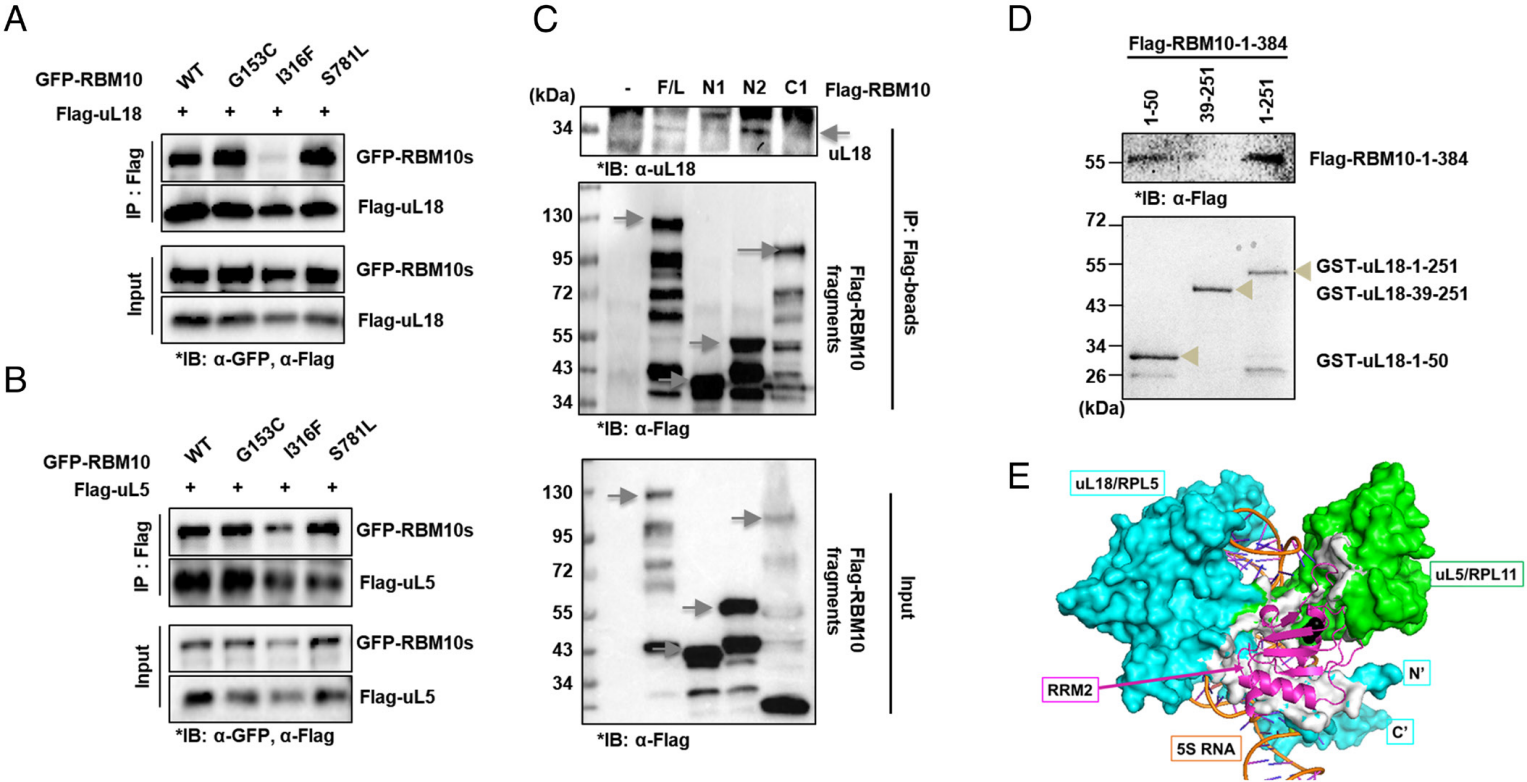
RBM10-I316F fails to bind to uL18 and uL5. (*A* and *B*) The binding between exogenous RBM10-I316F and Flag tagged uL18 or uL5 was decreased. HCT116^p53−/−^ cells were cotransfected with plasmids encoding GFP-tagged RBM10, RBM10-G153C, RBM10-I316F, or RBM10-S781L with Flag-uL18 (*A*) or Flag-uL5 (*B*) as indicated followed by co-IP-IB assays using antibodies as indicated. (*C*) Flag-RBM10 fragments were overexpressed in HCT116^p53−/−^ cells. The co-IP assay was followed using Flag-beads to detect the binding with endogenous uL18. IB assay was performed with indicated antibodies. (*D*) Flag-RBM10-1-384 fragment was overexpressed in H1299 cells. The cells were lysed and pulled down with GST-uL18 fragment proteins (1-50 aa, 39-251 aa, and 1-251 aa) generated using *E. Coli* after being checked for protein expression levels by Coomassie blue. The pulled-down RBM10 fragment 1-384 protein expression level was detected using Flag antibody by IB. (*E*) The prediction of a 3D model for the RBM10 complex with the 5S RNP was made at the GalaxyWEB server. The RRM2 region of RBM10 was docked to the uL18/uL5/5s rRNA portion of the cryo-EM structure of human ribosome (PDB 6zp). The predicted RRM2-binding site is highlighted in white.

Next, to elucidate the mechanism underlying the failure of RBM10-I316F in suppressing c-Myc expression, we analyzed three missense mutations (G153C, I316F, and S781L) of RBM10 for their ability to bind to uL18 and uL5 because these ribosomal proteins are required for RBM10 to suppress c-Myc (Fig. 4 *H* and *J*). These mutant RBM10s were previously predicted to be tumorigenic by the FATHMM algorithm used in the COSMIC database (40). Two of the mutants, S781L and I316F, failed to suppress lung cancer growth in vitro (40). In our effort to evaluate the impact of these mutants on the RBM10’s ability to bind to the RPs, we cotransfected GFP-RBM10s (wild type, G153C, I316F, or S781L) and Flag-uL18 in HCT116^p53−/−^ cells and conducted a set of co-IP-IB assays. Interestingly, only RBM10-I316F failed to be coimmunoprecipitated with uL18, whereas the other RBM10 mutants still bound to uL18 as did wild-type RBM10 (Fig. 6*A*). Notably, RBM10-I316F also failed to bind to uL5, though to a less degree (Fig. 6*B*). The residual binding of uL5 with RBM10 might be through endogenous uL18. These results suggested that uL18 and/or uL5 might bind to the RRM2 domain of RBM10 (Fig. 5*A*). Since RBM10-I316F almost completely lost its ability to bind to uL18 (Fig. 6*A*), but not uL5 (Fig. 6*B*), we speculated that uL18 is the major ribosomal protein that binds to this region. To test this idea, we decided to map the uL18-binding domain of RBM10 by performing co-IP-IB assays after introducing different Flag-tagged RBM10 fragments into HCT116^p53−/−^ cells (Fig. 6*C*). As a result, we found that the N2 region of RBM10 (Fig. 2*H*), which harbors the RRM2 domain, can bind to endogenous uL18 (Fig. 6*C*). To definitively determine whether this RRM2 domain is the uL18-binding domain and to map the RBM10-binding domain of uL18, we performed a GST-fusion protein-protein interaction assays with purified GST-uL18 protein fragments after transfecting HCT116^p53−/−^ cells with the Flag-RBM10-1-384 fragment. As shown in Fig. 6*D*, the N terminus of uL18 bound to the RBM10 RRM2 domain. Based on these data, we predicted a 3D model of RBM10 in a complex with the RPs (Fig. 6*E*). RRM2 could successfully dock into the 5S RNP complex by binding to uL18 N terminus strongly and associating with uL5 through weaker interactions. Taken together, these results demonstrate that uL18 via its N terminus with uL5 bind to the RBM10 via its RRM2 domain, and I316 is a key residue for this interaction as RBM10-I316F fails to bind to uL18 and to suppress c-Myc expression. All of these lead to the impairment of this mutant RBM10’s ability to suppress c-Myc activity (Fig. 5).

### RBM10-I316F Fails to Suppress Cancer Cell Proliferation and Tumorigenesis.

**Fig. 7.**
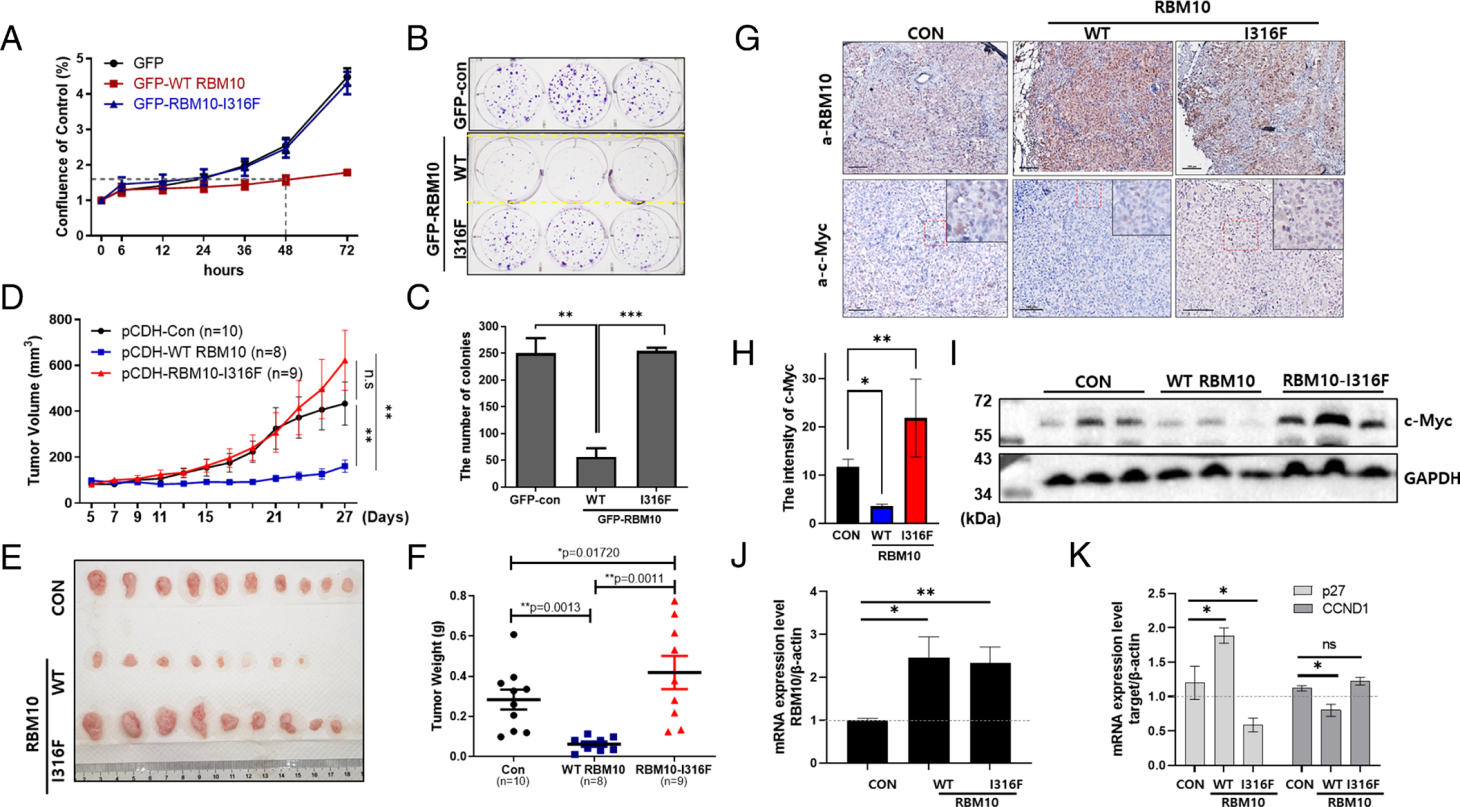
RBM10-I316F fails to reduce cancer cell proliferation and tumor growth. (*A*–*C*) H1299 cells were transfected with GFP-tagged control, RBM10, or RBM10-I316F for 36 h which has G418 resistance. The cells were treated with G418 to be selected for 16 h. The cells were reseeded onto 96-well for Incucyte detection to monitor proliferation rate over time (*A*). At the same time, the cells were reseeded onto six-well plates for colony formation assays (*B*), and the number of colonies was counted (*C*). The colonies were incubated for 10 d, fixed, and stained with crystal violet. (*D*) The average tumor volumes were measured at the indicated time points every other day for 27 d after pCDH-control, pCDH-RBM10, or pCDH-RBM10-I316F expressing lentivirus infected H1299 cancer cells were implanted into mice subcutaneously. The data are plotted as mean ± SE. Final volumes were analyzed for *P* value. **P < 0.01 (*D*). (*E* and *F*) Tumors were harvested 27 d after implantation. After tumors were isolated, representative images were taken (*E*), and the tumor weights were measured immediately (*F*). For panels *C*, *D*, and *F*, the Student’s two-tailed *t* test was used to determine the mean difference among groups. (*G*) RBM10 and c-Myc were stained in tumor tissue sections after harvested, fixed, and embedded. (Scale bar, 100 μm.) (*H*) The bar graph of the intensity of c-Myc for panel *G* (n = 6). Data are means ± SD. *P < 0.05 and **P < 0.01 (one-way ANOVA with Tukey’s multiple comparison tests). (*I*) c-Myc expression level in xenograft tumor lysate samples was confirmed by IB. GAPDH was used as loading control. (*J*) RBM10 mRNA levels were detected by qRT-PCR after RNA was extracted from xenograft samples. The expression levels were normalized to β-actin [n = 6 for pCDH-control group; n = 4 for pCDH-RBM10; n = 7 for pCDH-RBM10 (I316F)]. (*K*) The mRNA levels of p27 and CCND1 were measured using qRT-PCR after RNA was extracted from xenograft samples. β-actin was used as loading control. For panels *J* and *K*, the Student’s two-tailed *t* test was used to determine the mean difference among groups.

Previously, RBM10-I316F was shown to lose its ability to suppress proliferation of cultured lung cancer A549 cells (40). Since A549 cells contain wild-type p53, and RBM10 was recently shown to suppress cancer cell proliferation by activating p53 (20, 33), we wanted to see whether RBM10 might affect cancer cell proliferation independent of p53 status by comparing wild-type and mutant RBM10. As shown in Fig. 7 *A*–*C* and *SI Appendix*, Fig. S11 *A* and *B*, wild-type RBM10 overexpression in p53-deficient H1299 and HCT116^p53−/−^ cells was still able to suppress their survival and colony formation, whereas RBM10-I316F failed to do so. To test the effects of wild-type and mutant RBM10 on tumor growth in a more physiological context, we inoculated the p53-deficient H1299 cells into SCID mice after the cells were infected with lentivirus that expressed either wild-type RBM10 or RBM10-I316F (*SI Appendix*, Fig. S12 *A*–*D*). As expected (40–42), wild-type RBM10 when overexpressed drastically suppressed the growth of H1299 cells-derived xenograft tumors, whereas RBM10-I316F failed to do so (Fig. 7*D*). Remarkably, RBM10-I316F not only failed to suppress the tumor growth but also appeared to significantly promote the growth of xenograft tumors (Fig. 7 *E* and *F*). This is consistent with RBM10-I316F’s ability to enhance the serum-responsive elevation of endogenous c-Myc protein levels (Fig. 5*D*) and to bind to wild-type RBM10 (*SI Appendix*, Fig. S10). Consistent with these results, the c-Myc expression level was reduced in the wild-type RBM10 overexpressed xenograft tumors, but increased in the RBM10-I316F-bearing tumors (Fig. 7 *G*–*I*). As shown in Fig. 7 *J* and *K*, c-Myc’s downstream target genes in xenograft samples were modulated by the status of RBM10. These results not only demonstrate that the cancer-derived mutant RBM10-I316F loses its ability to suppress c-Myc and consequently to inhibit tumor growth but also suggest that this mutant might gain an ability to promote cancer growth potentially by inhibiting its wild-type counterpart.

## Discussion

It is known that c-Myc promotes proliferation and survival of cancer cells in part by boosting their ribosomal biogenesis. Previously, we showed that uL18 and uL5 can cooperatively inactivate c-Myc through direct interaction with the c-Myc protein in a negative feedback fashion (8, 34). However, it remains unaddressed whether the RPLs might work with other molecules in regulation of c-Myc stability and activity or not. Here, we reported that RBM10 acts as a regulator of c-Myc to reduce c-Myc stability and activity by partnering with the two ribosomal proteins uL18 and uL5, consequently suppressing the proliferation of human lung cancer cells and the growth of their xenograft tumors. Our findings are not only in line with a recent cell-based study showing that doxorubicin treatment induces RBM10 and cleaved-PARP while reducing c-Myc protein level (32) but also unveil the molecular mechanism underlying the anticancer role of RBM10, i.e., inhibiting c-Myc activity by working with uL18 and uL5 (Fig. 4).

First, we showed that RBM10 overexpression decreases c-Myc protein levels whereas knockdown of RBM10 induces the c-Myc protein levels (Figs. 1–3). These reversible effects were evident even when cancer cells were cultured under serum stimulation conditions (Fig. 1 *E* and *F*), indicating that serum-responsive c-Myc activation is also inhibited by RBM10. This inhibition appeared to be executed via their direct interaction (Fig. 2). However, RBM10 did not appear to interfere with the c-Myc-MAX binding (*SI Appendix*, Fig. S7), as it binds to the central domain of c-Myc, prior to the MAX-binding domain, via its own C-terminal domain (Fig. 2 *F* and *G*), suggesting that RBM10 might utilize a different mechanism to suppress c-Myc activity. Indeed, RBM10 can regulate the stability of c-Myc protein, as its overexpression reduced c-Myc half-life (Fig. 3 *A* and *B*) by enhancing c-Myc ubiquitination and degradation likely mediated by Fbw7α (Fig. 3*E* and *SI Appendix*, Fig. S8), whereas its knockdown increases c-Myc half-life (Fig. 3 *C* and *D*) by inhibiting c-Myc ubiquitination (Fig. 3*F* and *SI Appendix*, Fig. S8*B*).

Interestingly, our further studies showed that RBM10 requires uL18 and uL5 to regulate c-Myc stability and activity. First, we showed that exogenous and endogenous RBM10s can bind to both the RPLs in the nucleus (Fig. 4 *A*–*F*). Also, overexpression of Flag-uL18 (Fig. 4*G*) or Flag-uL5 (Fig. 4*I*) enhanced the reduction of c-Myc protein level by RBM10, while knockdown of endogenous uL18 (Fig. 4*H*) or uL5 (Fig. 4*J*) impeded this reduction. Therefore, these results strongly indicate that RBM10 regulates c-Myc via the ribosomal proteins. Interestingly, the cancer-derived RBM10-I316F mutant fails to reduce the protein level and activity of c-Myc (Figs. 5 and 7 *G–K*); instead, this RBM10 mutant appeared to augment the c-Myc level and activity (Figs. 5 and 7 *G*–*K*). As a result, RBM10-I316F significantly promoted proliferation of lung cancer cells and the growth of the cells-derived xenograft tumors in mice (Fig. 7 *A*–*F*). Remarkably, this RBM10-I316F mutant lost its ability to bind to uL18 and uL5 to less of a degree (Fig. 6 *A* and *B*). Since RBM10 via its N-terminal domain (aa 1-384) bound to uL18 at its N-terminal domain (aa1-50) (Fig. 6 *C* and *D*), we speculated that RBM10 can bind to uL18 directly and to uL5 indirectly via uL18 in the presence of 5s rRNA (Fig. 6*E* and *SI Appendix*, Fig. S9). The residual RBM10-bound uL5 molecules could be interacting via endogenous uL18 because uL18 and uL5 can form a complex via 5S RNA as previously shown (43, 44) and in Fig. 6*E* and *SI Appendix*, Fig. S9. Also, our recently published study showed that RBM10 is pulled down with anti-uL18 antibodies as detected by mass spectrometric analysis (19). Taken together, these results demonstrate that wild-type RBM10, but not RBM10-I316F, can inhibit c-Myc expression and activity, consequently suppressing the proliferation and growth of lung cancer cell and tumorigenesis. Our findings also suggest that the cancer-derived RBM10-I316F could promote cancer cell proliferation and survival by potentially suppressing the activity of its wild-type counterpart. This is an enticing question for our future exploration.

Previous studies showed that RBM10 possesses anticancer activity by regulating alternative splicing of several cancer-relevant genes (24). However, this does not appear to be a mechanism for RBM10 inhibition of c-Myc activity, as RBM10 had no effect on the alternative splicing of c-Myc RNA (*SI Appendix*, Fig. S5). Instead, our studies as described above reveal a mechanism for its anticancer role, i.e., to inhibit c-Myc activity by partnering with uL18 and uL5. Further dissection of this mechanism would provide more insights into whether the c-Myc inhibitory functions of RBM10 might cross talk with the splicing regulation of this tumor suppressor protein. Also, creating a genetically modified mouse model system would be helpful for our better understanding of the biological meaning of this tumor suppressor protein and its relationship with c-Myc. These lines of information would certainly be useful for our anticancer drug discovery in the future.

## Materials and Methods

### Plasmids and Antibodies.

The RBM10 expression plasmid was kindly provided by Juan Valcarcel (Centro de Regulación Genómica, Spain). The GFP-RBM10 (WT, G153C, I316F, and S781L) were kindly provided by Yongbo Wang (Fudan University, China). Flag-pcDNA3.1-RBM10, Flag-pcDNA3.1-RBM10 fragments plasmids, GST-pGEX-4T-1-RPL5/uL18 and GST-pGEX-4T-1-RPL5/uL18 fragments were constructed in our laboratory. The plasmids encoding HA-c-Myc fragments were kindly provided by Ping Wang (East China Normal University, China). The Flag-uL18, Flag-uL5, V5-c-Myc, HA-Ub, and His-Ub were described previously (8, 16, 34, 45–47). Anti-Flag (Sigma-Aldrich, catalogue no. F1804, diluted 1:3,000), anti-HA (Proteintech, catalogue no. 66006-1-Ig, diluted 1:3,000), anti-GFP (B-2, Santa Cruz Biotechnology, catalogue no. sc-9996, diluted 1:1,000), anti-RBM10 (Proteintech, catalogue no. 14423-1-AP, diluted 1:1,000 for IB) anti-RBM10 (Santa Cruz, catalogue no. sc-515548, diluted 1:100 for immunofluorescence), anti-c-Myc (Abcam, catalogue no. ab32072, diluted 1:100 for immunohistochemistry), anti-c-Myc (Proteintech, catalogue no. 10828-1-AP, diluted 1:1,000 for IB and 1:200 for immunofluorescence), anti-GAPDH (Proteintech, catalogue no. 60004-1-Ig, diluted 1:3,000), anti-Lamin A/C (E-1, Santa Cruz Biotechnology, catalogue no. sc-376248, diluted 1:1,000), and anti-β-actin (C4, Santa Cruz Biotechnology, catalogue no. sc-47778, diluted 1:3,000) were commercially purchased.

### Mouse Xenograft Experiments.

Twenty-seven SCID nude mice as xenograft models were divided into three groups to transplant cancer cells as control, wild-type RBM10, and RBM10-I316F groups. After wild-type RBM10 or RBM10-I316F was inserted into pCDH-CMV-MCS-EF1-Puro vector, the plasmids were used to generate lentivirus as described previously (48). pCDH-CMV-MCS-EF1-Puro vector was used as control. H1299 cells were infected with this lentivirus twice every other day for 4 d and incubated with 1 μg/mL of puromycin for 24 h to be selected. 5 × 10^6^ cells in 50 μL of PBS mixed with 50 μL of Matrigel (Corning, NY) were injected into SCID nude mice subcutaneously. The tumor area and the mice weight were monitored and recorded every other day, and the tumor volume was calculated as mm^3^ = length × (width)^2^ × 0.5. The ending point was decided in accordance with the National Institutes Health “Guide for the Care and Use of Laboratory Animals”. The tumors were harvested, and weight was measured and presented in histograms. This animal experiment was approved by the Institutional Animal Care and Use Committee at Tulane University School of Medicine.

### Immunoblotting and Immunoprecipitation.

Cells were harvested and lysed in lysis buffer consisting of 50 mM Tris/HCl (pH 7.5), 0.5% Nonidet P-40 (NP-40), 1 mM EDTA, 150 mM NaCl, 1 mM dithiothreitol (DTT), 0.2 mM phenylmethylsulfonyl fluoride (PMSF), 10 mM pepstatin A, and 1 mM leupeptin. Equal amounts of clear cell lysate (20–50 µg) were used for immunoblotting (IB) analyses. Immunoprecipitation (IP) was conducted using antibodies or antibody-tagged beads as indicated in the figure legends. Briefly, 300–1,000 µg of proteins were prewashed with Protein A and G beads (Santa Cruz Biotechnology). Then, the proteins were incubated with the indicated antibodies or HA- and Flag-beads at 4 °C for either 4 to 6 h or overnight. Protein A or G beads were then added into protein samples with only antibodies attached, and the mixture was incubated at 4 °C for additional 2 h. All of the beads attached samples were washed at least three times with lysis, SNNTE [50 mM Tris/HCl (pH 7.4), 5 mM EDTA, 5% sucrose, 1% NP-40, and 0.5 M NaCl], or RIPA [50 mM Tris/HCl (pH 7.4), 150 mM NaCl, 1% Triton X-100, 0.1% SDS, and 1% Na Deoxycholate] buffers. After washing, the samples were boiled with SDS loading buffer and resolved by 8–15% SDS-PAGE. Bound proteins were detected by IB with antibodies as indicated in the figure and figure legends. Protein markers were purchased from Fisher BioReagents and APExBIO.

### GST and His Fusion Protein Association Assay.

The GST and His pull-down assays were performed using the methods as described previously (49). Briefly, GST-tagged uL18/RPL5 fragments and His-tagged RBM10 proteins were expressed in *E. coli* and conjugated with either glutathione-Sepharose 4B beads (Sigma-Aldrich) or Ni-NTA agarose beads (Thermo Scientific). Protein–protein interaction assays were conducted by using cell lysates. After H1299 and HCT116^p53−/−^ cells were transfected with Flag-RBM10 fragment (1-384) and HA-c-Myc, the proteins were extracted using lysis buffer. Then, the lysates were incubated and gently rotated with GST-tagged uL18 fragments or His-tagged RBM10 for 1 to 2 h at RT or at 4 °C. The mixtures were washed three times with lysis buffer (50 mM Tris/HCl pH 8.0, 0.5% NP-40, 1 mM EDTA, 150 mM NaCl, and 10% glycerol). Bound proteins were analyzed by IB with the antibodies as indicated in the figure legends.

### Cell Fractionation.

Cell fractionation assay was performed as previously described (49). Briefly, approximately 1 × 10^6^ cells were collected, washed with PBS, and resuspended in CE buffer (10 mM HEPES, 60 mM KCl, 1 mM EDTA, 0.075% NP-40, 1 mM DTT, and 1 mM PMSF, adjusted to pH 7.6) for 10 min on ice to collect cytoplasmic extract. After washing, NE buffer (20 mM Tris/HCl, 420 mM NaCl, 1.5 mM MgCl2, 0.2 mM EDTA, 1 mM PMSF, and 25% glycerol, adjusted to pH 8.0) was used to isolate nuclei pellet. Phenylmethylsulfonyl fluoride (PMSF) and DTT were added freshly. Finally, the cytoplasmic extract and the nuclei pellet were spun down at maximum speed for 10 min.

### PCR Analysis for Splicing Effect.

To check the effect of RBM10 on *c-myc* gene splicing, total RNA was extracted from HCT116^p53−/−^ cells after transfection was performed. cDNA was synthesized from RNA, and RT-PCR was conducted using primers as described in Kazuyuki et al. (50). Primer sequences for c-Myc are Exon1-Exon3 (Forward: 5′-CAGGACCCGCTTCTCTGAAA-3′), Exon2-Exon3 (Forward: 5′-CGTTAGCTTCACCAACAGGA-3′), Reverse: 5′-TTACGCACAAGAGTTCCGTA-3′. Primer sequences for GAPDH (Forward: 5′-AGCCACATCGCTCAGACAC-3′, Reverse: 5′-AGCATCGCCCCACTTGATT-3′). For an RBM10 working indicator, the FAS gene was used as previously described (35).

## Supplementary Material

Appendix 01 (PDF)

Movie S1. Endogenous RBM10 and c-Myc locations were confirmed by immunofluorescence assay in HCT116^p53+/+^. The cell was zoomed and made as a 3D video in to see more accurate localization of RBM10 and c-Myc proteins. (RBM10: green, c-Myc: red, DAPI: blue)

Movie S2. Endogenous RBM10 and c-Myc proteins were confirmed by immunofluorescence assay in HCT116^p53-/-^ cells. The HCT116^p53-/-^ cell was zoomed and made as a 3D video using confocal microscopy. (RBM10: green, c-Myc: red, DAPI: blue)

Movie S3. Endogenous RBM10 and c-Myc expression levels were confirmed by immunofluorescence assay in the HCT116^p53-/-^ cell. Only RBM10 (green) and c-Myc (red) were detected to show more accurate localization.

## Data Availability

All data are included in the manuscript and/or *SI Appendix*.
