# Supplementary material for: RNA-binding motif protein 10 inactivates c-Myc by partnering with ribosomal proteins uL18 and uL5
Source: Proc Natl Acad Sci U S A. 2023 Nov 30;120(49):e2308292120. doi: 10.1073/pnas.2308292120 (PMC10710042; doi:10.1073/pnas.2308292120)
Supplement: Supplementary file 1 — Appendix 01 (PDF) [file pnas.2308292120.sapp.pdf]

Supplementary Information

RNA-Binding motif protein 10 inactivates c-Myc by partnering with ribosomal proteins uL18 and uL5

Hyemin Lee[1], Ji Hoon Jung[1, 2], Hyun Min Ko[1], Heewon Park[1], Allyson M. Segall[1, 3], Roger L. Sheffmaker[1, 4], Jieqiong Wang[1], Wesley D. Frey[1], Nathan Pham[1], Yongbo Wang[5], Yiwei Zhang[1], James G. Jackson[1],Shelya X. Zeng[1], and Hua Lu[1*]

[1]Department of Biochemistry and Molecular Biology and Tulane Cancer Center, Tulane University School of Medicine, New Orleans, Louisiana 70112, USA;

[2]College of Korean Medicine, Kyung Hee University, Seoul 02447, Republic of Korea (current address);

[3]Department of Neuroscience, Tulane University, New Orleans, Louisiana 70118, USA; [4]Department of Cell and Molecular Biology, Tulane University, New Orleans, Louisiana 70118, USA;

[5]Department of Cellular and Genetic Medicine, School of Basic Medical Sciences, Fudan University, Shanghai, 200032, China.

* Correspondence: Hua Lu, Department of Biochemistry and Molecular Biology, Tulane Cancer Center, Tulane University School of Medicine, 1430 Tulane Ave., New Orleans, Louisiana, 70112, USA.

Email: hlu2@tulane.edu

Author Contributions: H.M.L contributed ~ 65% of the data, while J.H.J, contributed ~30%, to this study by designing and performing the majority of the experiments as described in this manuscript under supervision of H.L; Also J.H.J initiated this study, and H.M.L completed the study and performed additional experiments to address reviewers' questions; H.M.K, A.M.S, R.L.S, J.Q.W, and N.P helped perform and assist in some of the in vitro and animal experiments; H.P generated a predicted 3D model; W.D.F (under mentorship of J.G.J) and S.X.Z assisted to generate new plasmids and lentivirus; Y.W. provided constructs for RBM10 mutants; Y.W.Z assisted in animal protocol; W.D.F and J.G.J assisted in the revision of manuscript; H.M.L, J.H.J, S.X.Z, and H.L. designed the study and analyzed the data; H.M.L and H.L designed the study and wrote and revised the revision of this manuscript.

Competing Interest Statement: The authors declare no conflict of interest.

Classification: Biological Sciences: Biochemistry, Cell Biology, Oncology

Keywords: RBM10, c-Myc, RPL5/uL18, RBM10 mutation, and lung cancer.

This PDF/DOC file includes:

Supplementary Figures 12

Materials and Methods

Cell culture and transient transfection

HCT116 $^{p53+/+}$ and HCT116 $^{p53-/-}$ cells were generous gifts from Dr. Bert Vogelstein at the John Hopkins Medical Institutes. H1299 cells were purchased from American Type Culture Collection (ATCC). No mycoplasma contamination was found. All cells were cultured in Dulbecco's modified Eagle's medium (DMEM) supplemented with 10% fetal bovine serum, $50Uml^{-1}$ penicillin and $0.1mgml^{-1}$ streptomycin. All cells were maintained at 37 °C in a 5% CO_2 humidified incubator. Cells were seeded on the plate overnight. Next day, cells were transfected with plasmids as indicated in figure legends using TurboFect transfection reagent (Thermo Scientific, Waltham, MA) following the manufacturer's protocol. Cells were harvested at 36–72 h post-transfection for future experiments.

Cell proliferation assay

Cells were seeded into 96-well plates at a density of 1,000 – 5,000 cells per well. Cell proliferation rate was detected every day and analyzed using IncuCyte S3 Live-Cell imaging system and the IncuCyte S3 Basic Analyzer software module (Essen Bioscience, MI).

Colony formation assay

1,000 Cells were plated onto 6-well plates. Fresh media was changed every 3-4 days until the colonies were visible. 800 µg/mL of Geneticin 418 was added for cell selection. Cells were fixed with paraformaldehyde and stained with crystal violet solution at RT.

Hematoxylin and Eosin Staining

The tissues were fixed in formalin, embedded in paraffin and sectioned at a thickness of 4 µm. The slides were stained with hematoxylin and eosin by following commercial protocol.

Immunohistochemistry

After the tumor tissues were fixed in formalin, the samples were embedded in paraffin and sliced as described above. The sliced samples were deparaffinized with heat and xylenes, and rehydrated using alcohol. Antigen retrieval was performed by boiling in 10 mM Sodium Citrate using microwave. After cooling down slowly, slides were blocked by incubating with blocking buffer (goat serum and glycine with 0.8 % Triton X-100 in PBS) for one hour at RT. Diluted primary antibodies in blocking buffer were incubated on slides overnight at 4 ℃. Before incubated with biotinylated rabbit secondary antibodies (Vector Laboratories, Inc. CA) diluted in blocking buffer for one hour at RT, endogenous peroxides were inactivated by adding 3% hydrogen peroxide for 10 m. Following that, secondary antibodies, ABC kits and DAB kits (Vector Laboratories) were used to develop. The samples were co-stained with hematoxylin, and then dehydrated and mounted in accordance with manufacturer instructions. The slides were examined under Akoya PhenoImager Fusion Slide Scanner and captured using Phenochart software 1.2 (AKOYA Biosciences, MA and CA).

Immunofluorescence staining

After cells were fixed in formaldehyde, the cells were permeabilized using 0.2 % Triton X-100 and blocked with 1% BSA in PBST (PBS with 0.1 % Tween 20). First primary antibodies were added into the samples and incubated at 4 °C overnight. The next day, the slides were incubated with secondary antibodies (Alexa-

488 or Alexa-594) (Invitrogen-Thermo, CA), for 1 hr at RT. Following this, another primary antibody was added and incubated for 2 hr at RT to co-stain. Another secondary antibody for the primary antibody was added and incubated for 1 hr at RT. The nuclei were stained with DAPI (Sigma). The slides were mounted and detected by Confocal microscopy (Nikon TiE-2, Nikon Inc., Tokyo, Japan). The green or red intensity of each cell was measured by Image J (NIH) one by one, and normalized by each picture's average intensity. The slope for overexpressed group was calculated as linear regression. p-value is significantly deviated from zero for overexpressed groups. Non-overexpressed groups have non-significant differences from zero. The slopes and the p-values were calculated by GraphPad Prism 9.4 software (GraphPad Software, CA, USA).

Reverse transcription and quantitative real time-PCR analysis

Total RNA was isolated from cells using Trizol (Invitrogen-Thermo, CA) following the manufacturer's protocol. Total RNAs of 2 μg were used as templates for reverse transcription using poly-$(T)_{20}$ primers and M-MLV reverse transcriptase (Promega, Madison, WI). Quantitative real time-PCR (RT-qPCR) was conducted using SYBR Green Mix (BioRad, Hercules, CA) according to the manufacturer's protocol. The primers are as follows: h-RBM10, (F)5'- CTCTACTATGACCCCAACTCCCA-3' and (R)5'-GTCCGCCTCTCCCCATCCCA-3'; h-MYC, (F)5'-CCACCAGCAGCGACTCTGA-3' and (R)5'-GCAGAAGGTGATCCAGACTC-3'; h-GAPDH, (F)5'- GATTCCACCCATGGCAAATTC-3' and (R)5'-AGCATCGCCCCACTTGATT-3'; h-CCND1, (F)5'- GGAGCTGCTGCAAATG-3' and (R)5'-GGAGGGCGGATTGGA-3'; h-TERT, (F)5'- ATGCGACAGTTCGTGGCTCA-3' and (R)5'-ATCCCCTGGCACTGGACGTA-3'; h-CDKN1B (p27), (F)5'- CCGGTGGACCACGAAGAGT-3' and (R)5'-GCTCGCCTCTTCCATGTCTC-3'; h-ATF4, (F)5'- TTCCTGAGCAGCGAGGTGT-3' and (R)5'-AGCCTTGTCGCTGGAGAAC-3'; h-BTG2, (F)5'- CCAGGAGGCACTCACAGAGCA-3' and (R)5'-ACCCACAGGGTCAGCTCGCT-3'; h-5s rRNA, (F)5'-GGCCATACCACCCTGAACGC-3' and (R)5'-CAGCACCCGGTATTCCCAGG-3 (1); h-β-actin, (F)5'- CATGTACGTTGCTATCCAGGC-3' and (R)5'-CTCCTTAATGTCACGCACGAT-3'.

RNA interference

For RNA interference, 40-60 nM of 5s rRNA-siRNA (5′-GGGAAUACCGGGUGCUGUAUU-3′), RBM10-siRNA (Sigma, catalogue no. SASI_Hs01_00199550) or negative control siRNA (5'-UUCUCCGAACGUGUCACGU-3') were introduced into cells using TurboFect transfection reagent (Thermo Scientific) following the manufacturer's protocol. Cells were harvested 48-72 h after transfection for next experiments, such as Immunoblot (IB), co-immunoprecipitation-immunoblot (co-IP-IB) or RT-qPCR.

***In vivo* ubiquitination assay.**

In vivo ubiquitination assays were performed as previously described (2). Shortly, HCT116 $^{p53-/-}$ cells were transfected with the plasmids for 48-72 hr as indicated in the figures and figure legends. After the transfection, MG132 (20-40 μM) was added into the cells for 4-6 hr. Ubiqutinated c-Myc protein levels were determined by IB analysis with α-c-Myc after conjugation with HisPur™ Ni-NTA Resin (Thermo Scientific).

Statistical analysis

All in vitro experiments were performed in biological triplicate. The student's two-tailed *t*-test and one-way ANOVA with Tukey's multiple comparison tests were used to determine mean difference among groups. $P < 0.05$ was considered statistically significant. Data are presented as mean ±SD or ±SE.

Sup 1

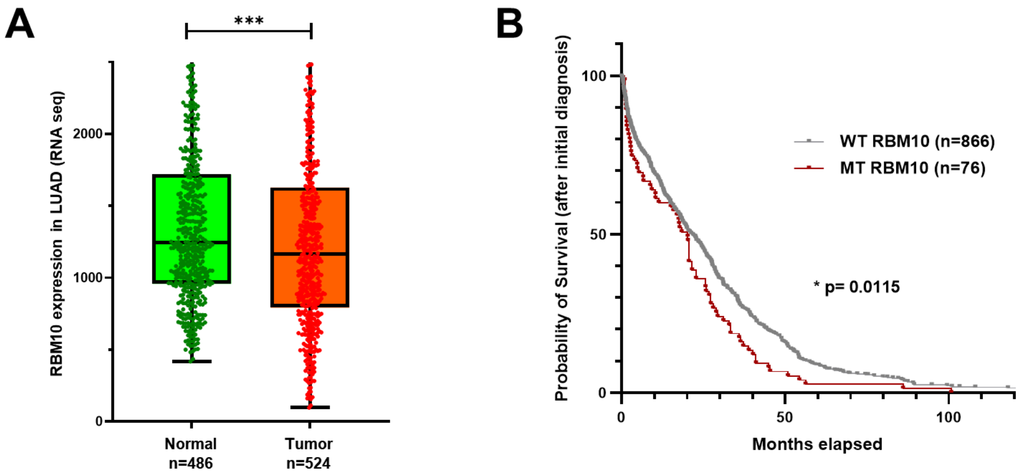

Supplementary Figure 1. A. A TN (Tumor vs. Normal) plot for the comparison of RBM10 expression in normal and tumor tissues from patient samples. ***P < 0.001. **B.** The Kaplan–Meier plot rate from MSK study in Nature Medicine 2022 showed the metastatic non-small cell lung cancer patients with mutated RBM10 survival rate was worse than RBM10 wild type patients. Log-rank (Mantel-Cox) test was performed for p-value. Structural variants of RBM10 were selected for the mutated RBM10 NSCLC patients.

Sup 2

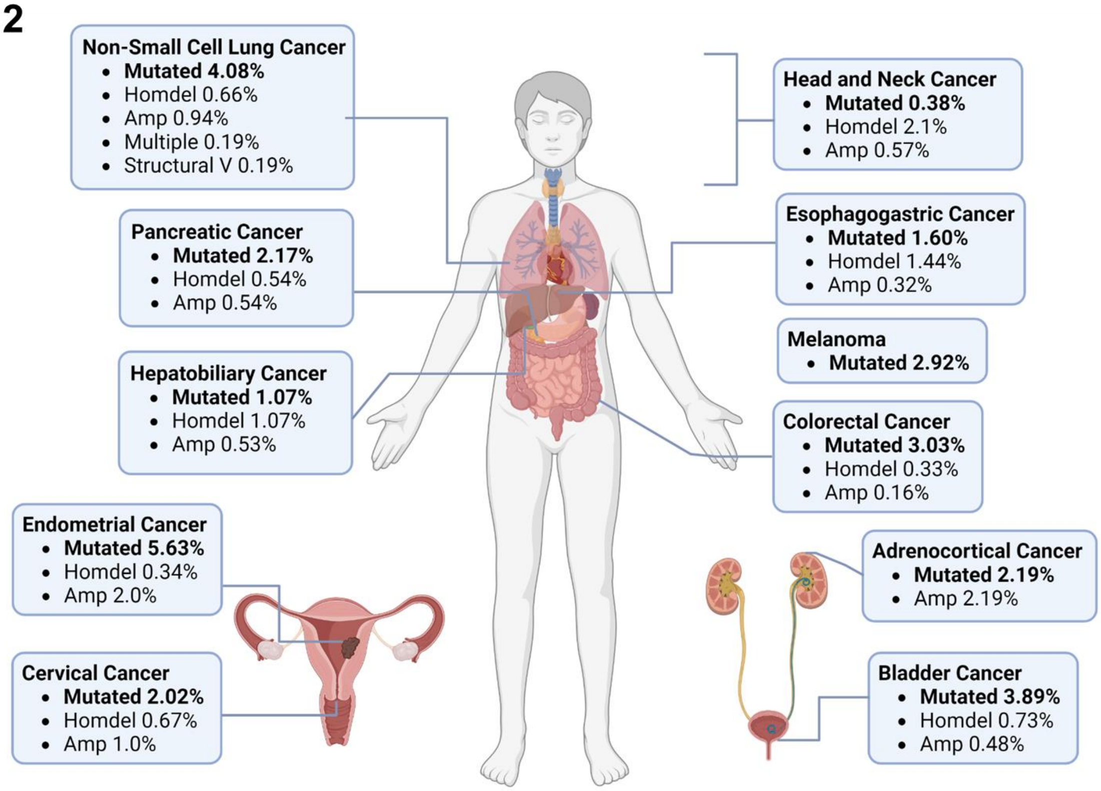

Supplementary Figure 2. The RBM10 alteration type and frequency were described. Data was downloaded from cBioportal (http://www.cbioportal.org/) and reformed as shown using BioRender. A total of 10,967 samples in TCGA PanCancer Atlas Studies (3, 4) were queried.

Sup 3

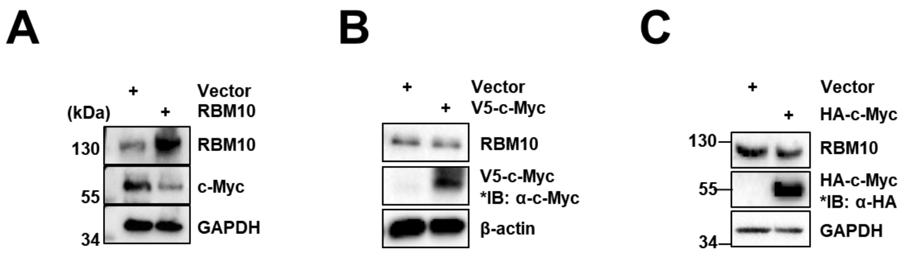

Supplementary Figure 3. A. c-Myc expression level was confirmed by IB after RBM10 was overexpressed in H1299 cells. **B.** HCT116$^{p53-/-}$ cells were transfected with control vector or V5-c-Myc, and the protein expression levels were detected by IB. **C.** Control vector or HA-c-Myc was transfected into H1299 cells, and the protein levels were confirmed by IB with indicated antibodies. GAPDH and β-actin were used as loading controls.

Sup 4

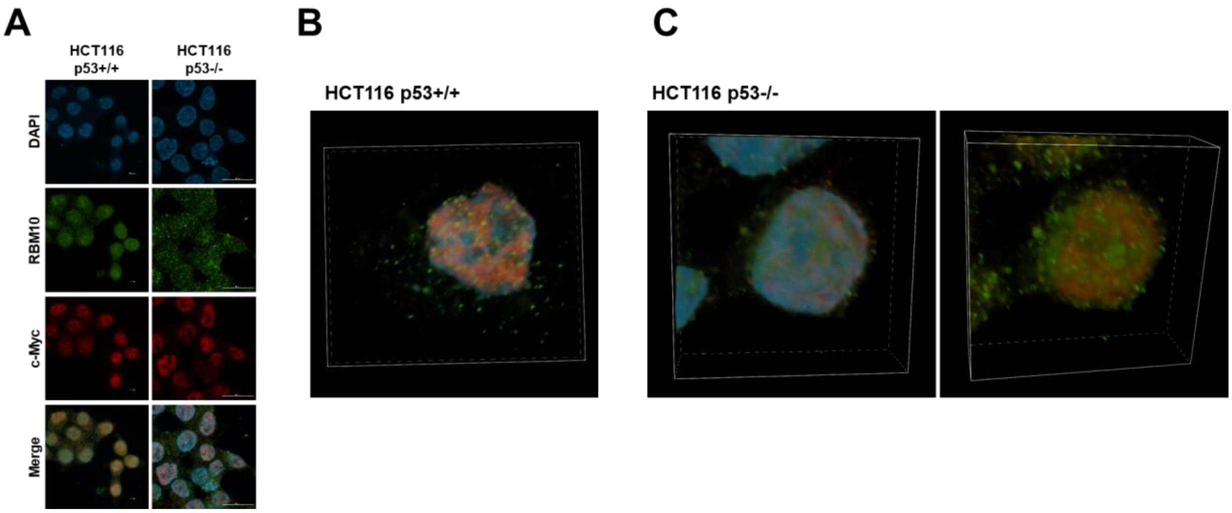

Supplementary Figure 4. Endogenous RBM10 and c-Myc expression levels were detected by immunofluorescence assay in HCT116$^{p53+/+}$ and HCT116$^{p53-/-}$ cells. After the cells were fixed, permeabilization, blocking, staining of α-RBM10 and α-c-Myc followed by secondary antibodies were performed. **A.** The cells were imaged in their respective channels. Scale bar: 20 μm. **B and C.** HCT116$^{p53+/+}$ (**B**) and HCT116$^{p53-/-}$ cells (**C**) were zoomed and made as 3D videos in to see more accurate localization of RBM10 and c-Myc proteins (green for RBM10, red for c-Myc, and blue for DAPI in the images and videos). **(Videos are separately uploaded as Movie S1, S2 and S3.)**

Movie S1. Endogenous RBM10 and c-Myc locations were confirmed by immunofluorescence assay in HCT116$^{p53+/+}$. The cell was zoomed and made as a 3D video in to see more accurate localization of RBM10 and c-Myc proteins. (RBM10: green, c-Myc: red, DAPI: blue)

Movie S2. Endogenous RBM10 and c-Myc proteins were confirmed by immunofluorescence assay in HCT116$^{p53-/-}$ cells. The HCT116$^{p53-/-}$ cell was zoomed and made as a 3D video using confocal microscopy. (RBM10: green, c-Myc: red, DAPI: blue)

Movie S3. Endogenous RBM10 and c-Myc expression levels were confirmed by immunofluorescence assay in the HCT116$^{p53-/-}$ cell. Only RBM10 (green) and c-Myc (red) were detected to show more accurate localization.

Sup 5

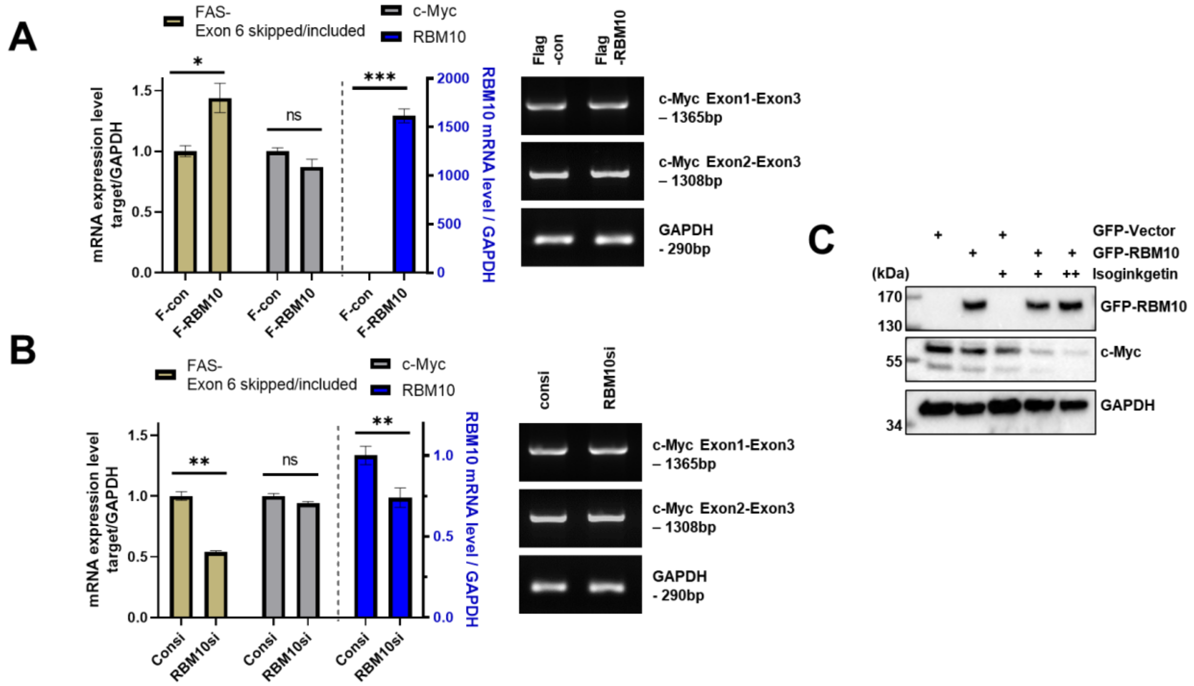

Supplementary Figure 5. RBM10 does not alter splicing of c-Myc mRNA. **A and B. (Left graph panels)** Fas(CD95), c-Myc and RBM10 mRNA levels were measured by qRT PCR after RBM10 overexpression (A) or knockdown (B) in HCT116$^{p53-/-}$ cells. Fas was used as an indicator of alternative splicing by RBM10 as described previously (5). **A and B (Right panels).** Agarose Gel Electrophoresis was performed to detect splicing alterations from RBM10 on c-Myc in HCT116$^{p53-/-}$ cells. **C.** c-Myc expression level was checked by IB following overexpression of GFP-RBM10 and treatment with Isoginkgetin (20 or 40 μM) in H1299 cells.

Sup 6

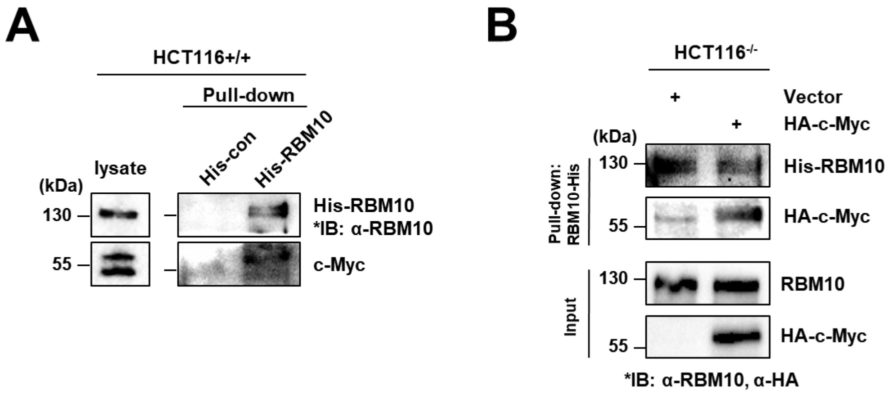

Supplementary Figure 6. c-Myc protein was immunoprecipitated by His-RBM10 beads. His-tagged RBM10 beads were generated from *E. coli*. **A.** HCT116$^{p53+/+}$ cells lysate was incubated with either His-control or His-RBM10 beads. **B.** HCT116$^{p53-/-}$ cells were treated with either control vector or HA-c-Myc overexpression vector, and the cells were harvested and lysed. The lysates were pulled down using His-RBM10 beads. The beads and lysate samples were incubated for 3 h at 4 ℃ and additional 40 m at RT followed.

Sup 7

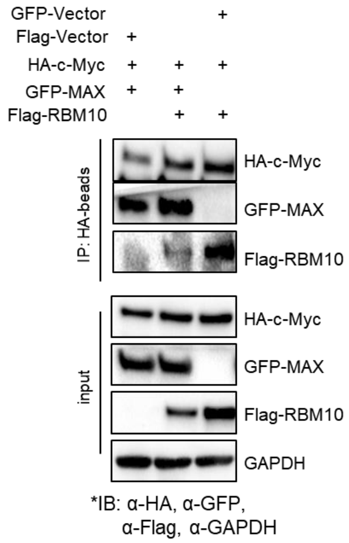

Supplementary Figure 7. MAX disrupts the binding of c-Myc and RBM10. After HCT116$^{p53-/-}$ cells were co-transfected with control vectors, HA-c-Myc, GFP-MAX and Flag-RBM10 as indicated, the lysates were pulled down using HA-beads. The bound proteins with HA-tagged c-Myc and input protein expression levels were confirmed by IB.

Sup 8

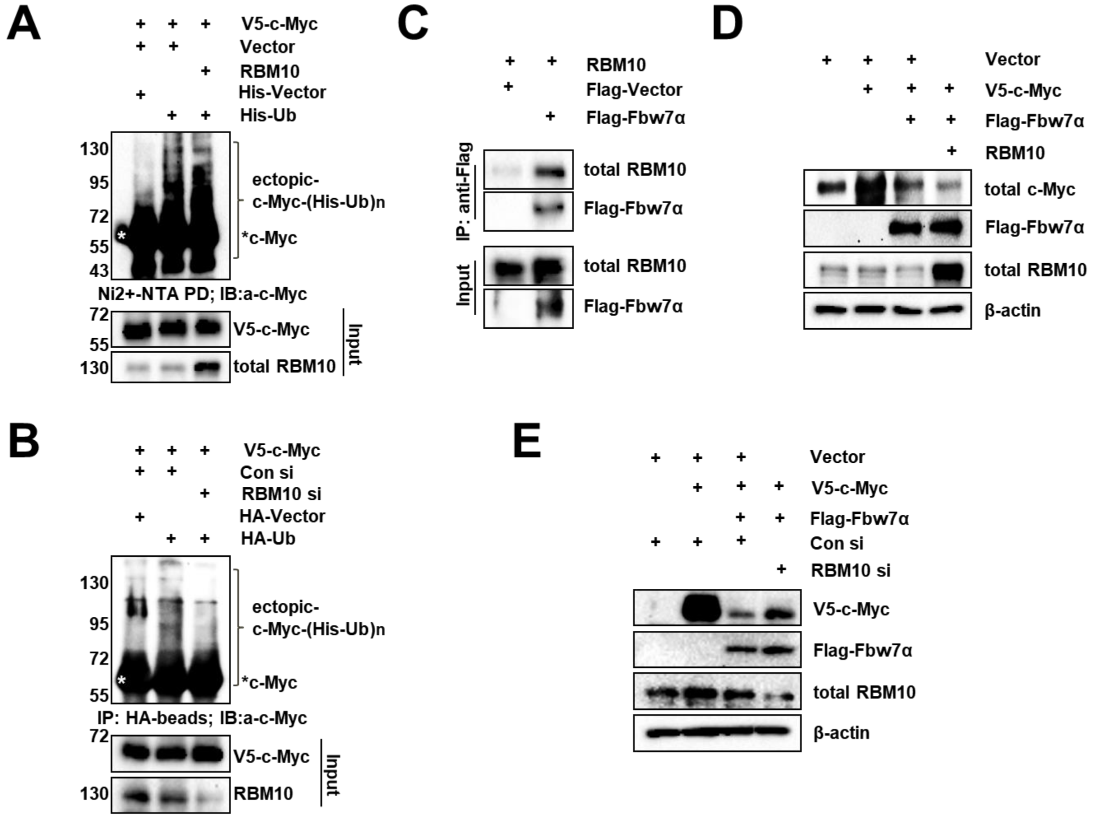

Supplementary Fig 8. RBM10 regulates exogenous c-Myc ubiquitination with Fbw7 synergistically. **A.** HCT116$^{p53-/-}$ cells were transfected with combinations of plasmids encoding control vectors, V5-c-Myc, His-Ub and RBM10 for overexpression respectively. Six hours before being harvested for an ubiquitination assay, the cells were treated with MG132 (20 μM). **B.** HCT116$^{p53-/-}$ cells were transfected with control or RBM10 siRNA plus the combinations of plasmids encoding control vector, V5-c-Myc or HA-Ub, and treated with MG132 (20 μM) for 6 h before being harvested for an ubiquitination assay. For both panels A and B, IB analysis using anti-c-Myc and anti-RBM10 antibodies was performed to detect bound and input proteins. **C.** After HCT116$^{p53-/-}$ cells were transfected with plasmids as indicated, co-IP assay was performed using α-Flag followed by IB. α-RBM10 and α-Flag were used for IB detection. **D.** HCT116$^{p53-/-}$ cells were transfected with control vector, V5 tagged c-Myc, Flag tagged Fbw7α, RBM10 for 48 h, harvested and lysed for IB detection. α-c-Myc, α-Flag. α-RBM10 and α-β-actin were used for IB detection. **E.** After control or RBM10 siRNA was introduced into HCT116$^{p53-/-}$ cells, control vector, V5 tagged c-Myc, Flag tagged Fbw7α were overexpressed as indicated. The cells were collected and lysed for IB detection using α-V5, α-Flag, α-RBM10 and α-β-actin. pcDNA empty vector was used to keep plasmid DNA concentration equivalent added to other samples. β-actin was used as loading control for **D and E**.

Sup 9

A

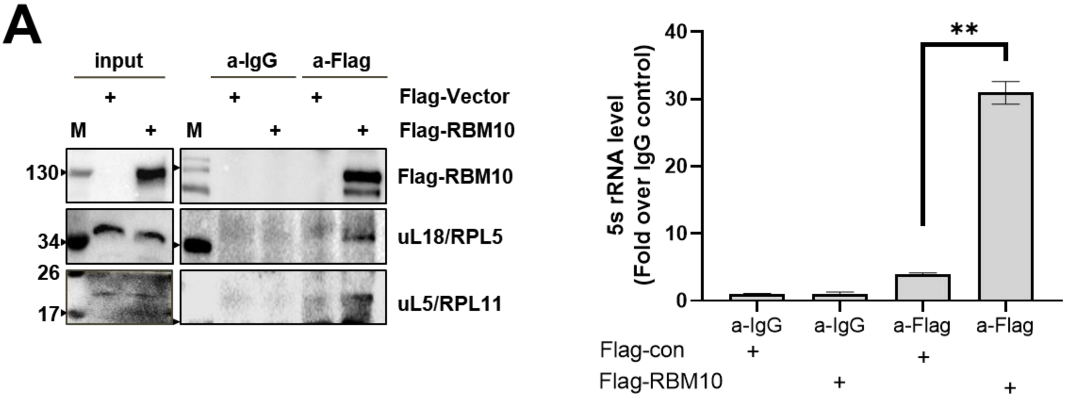

B

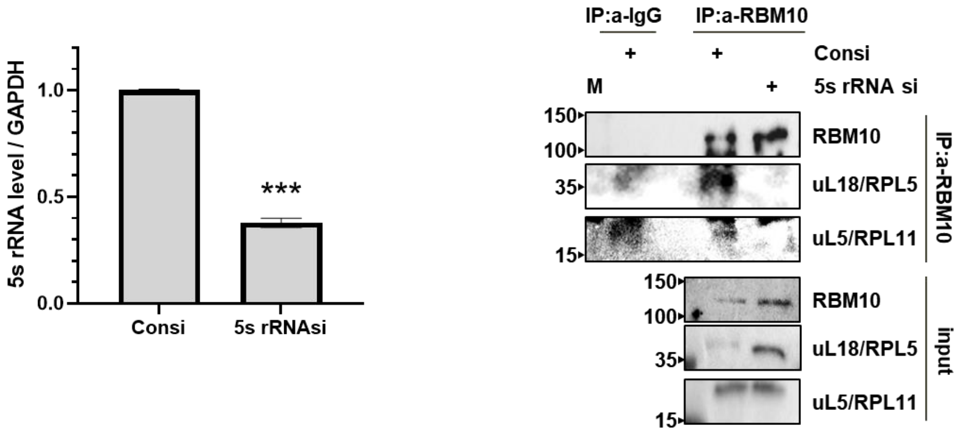

Supplementary Fig 9. 5s rRNA is a component of the complex of RBM10/uL18/uL5 in HCT116$^{p53-/-}$ cells. **A.** After HCT116$^{p53-/-}$ cells were transfected with either Flag tagged control vector or RBM10, the lysates were pulled down with anti-IgG or anti-Flag and detected by IB using α-Flag, α-uL18 and α-uL5 (Left). 10% of these co-IP samples were used for RNA isolation to detect 5s rRNA level. cDNA was synthesized and then qRT-PCR was performed (Right). **B.** After control or 5s rRNA siRNA was treated in HCT116$^{p53-/-}$ cells, 5s rRNA level was measured by qRT-PCR (Left) to confirm knockdown efficacy. The cells were then lysed and co-IP-IB analysis followed (Right). For pull-down, IgG or RBM10 antibody was used. α-RBM10, α-uL18 and α-uL5 were used for IB detection.

Sup 10

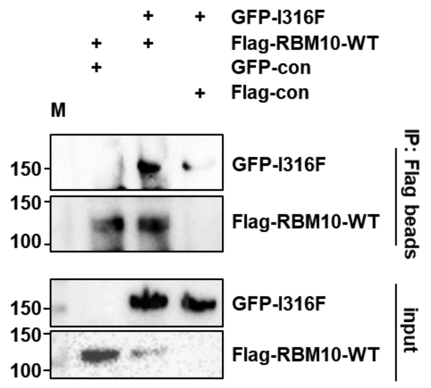

Supplementary Fig 10. Ectopic RBM10-I316F binds to ectopic RBM10. HCT116$^{p53-/-}$ cells were treated with GFP tagged control vector or RBM10-I316F and Flag tagged control vector or RBM10 as indicated. After 48 h, cells were collected and lysed for subsequent co-IP-IB assays. Flag-beads were used to pull down Flag-RBM10 binding proteins. α-GFP and α-Flag were used for IB detection.

Sup 11

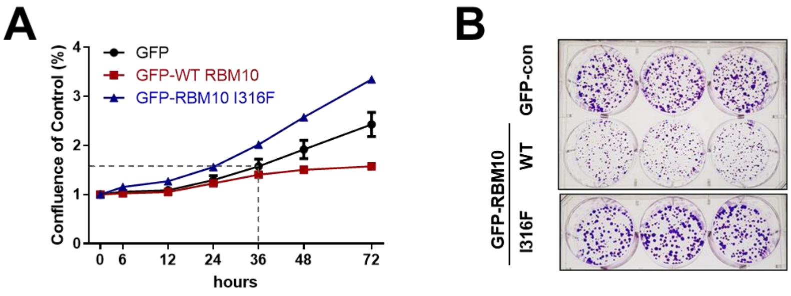

Supplementary Fig 11. RBM10-I316F fails to reduce colon cancer cell proliferation. HCT116$^{p53-/-}$ cells were transfected with GFP tagged control, RBM10 or RBM10-I316F for 36 h and selected with G418 treated for following 16 h. The cells were re-seeded onto 96-well for Incucyte detection to check proliferation rate overtime **(A)**. At the same time, the cells were re-seeded onto 6-well plates for colony formation assays and incubated for 10 days **(B)**. The cells were fixed and stained with crystal violet.

Sup 12

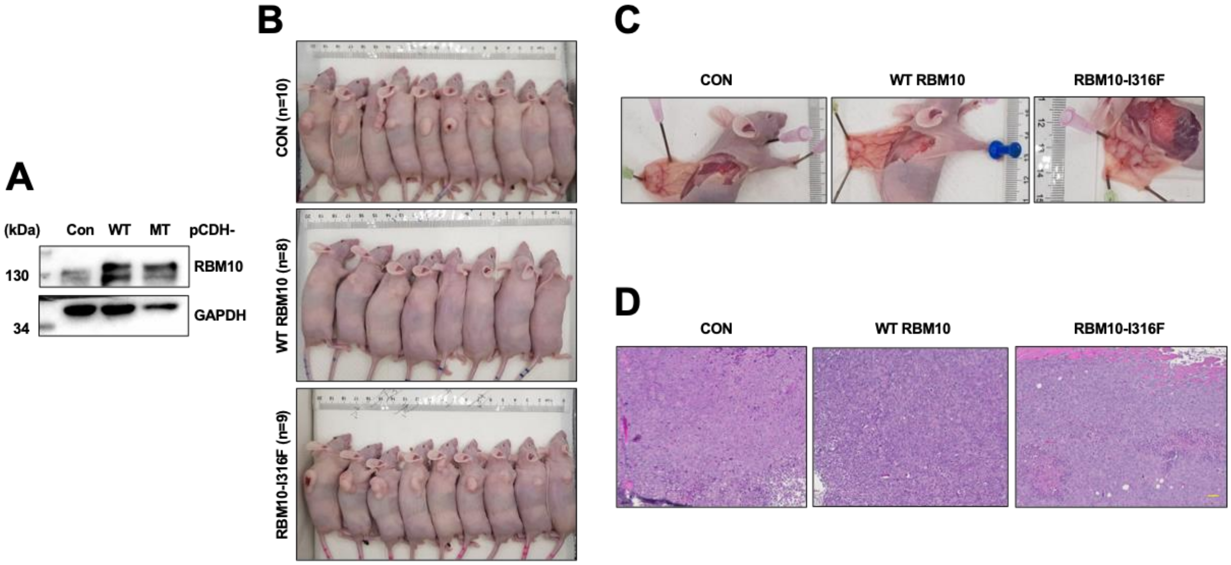

Supplementary Figure 12. RBM10-I316F showed more growth in xenograft models. **A.** Stable cell lines overexpressing either RBM10 or RBM10-I316F (MT) protein, as compared to control, was confirmed by IB analysis. The cells were subcutaneously injected into nude mice to generate xenograft tumors. **B.** The photographs of the mice that harbor xenograft tumors before tumors were harvested. **C.** The representative images of the tumors injected into mice subcutaneously. **D.** H&E staining was performed after the xenograft tumors were harvested, fixed and embedded. Scale bar; 50 μm.

References

1. A. G. Winter *et al.*, RNA polymerase III transcription factor TFIIIC2 is overexpressed in ovarian tumors. *Proc Natl Acad Sci U S A* **97**, 12619-12624 (2000).
2. J. Wang *et al.*, Valosin-Containing Protein Stabilizes Mutant p53 to Promote Pancreatic Cancer Growth. *Cancer Res* **81**, 4041-4053 (2021).
3. E. Cerami *et al.*, The cBio cancer genomics portal: an open platform for exploring multidimensional cancer genomics data. *Cancer Discov* **2**, 401-404 (2012).
4. J. Gao *et al.*, Integrative analysis of complex cancer genomics and clinical profiles using the cBioPortal. *Sci Signal* **6**, pl1 (2013).
5. A. Inoue *et al.*, RBM10 regulates alternative splicing. *FEBS Lett* **588**, 942-947 (2014).
